# Molecular elucidation of brain lipofuscin in aging and Neuronal Ceroid Lipofuscinosis

**DOI:** 10.21203/rs.3.rs-6010379/v1

**Published:** 2025-03-19

**Authors:** Sofia Massaro Tieze, Alexander Esqueda, Rachel McAllister, Matija Lagator, Betül Yücel, Eric Sun, TuKiet T. Lam, Nicholas Lockyer, Kallol Gupta, Sreeganga S. Chandra

**Affiliations:** 1 Departments of Neurology & Neuroscience, Yale University, New Haven, CT, USA; 2 Interdepartmental Neuroscience Program, Yale University, New Haven, CT, USA; 3 Department of Cell Biology, Yale University, New Haven, CT, USA; 4 Photon Science Institute, Department of Chemistry, University of Manchester, Manchester, UK; 5 Rosalind Franklin Institute, Rutherford Appleton Laboratory, Didcot, Oxfordshire, UK; 6 Departments of Molecular Biophysics and Biochemistry, Yale University, New Haven, CT, USA; 7 Keck Mass Spectrometry & Proteomics Resource, W.M. Keck Biotechnology Resource Laboratory, New Haven, CT, USA; 8 Yale College, Yale University, New Haven, CT, USA; 9 Senior author; 10 Lead contact

## Abstract

Lipofuscin is an autofluorescent material that accrues in brain tissues with age and in Neuronal Ceroid Lipofuscinosis (NCL), a neurodegenerative disease with pediatric onset. The distribution, composition, and organellar origin of lipofuscin have remained unclear despite its widespread presence in aged tissues and involvement in neurodegeneration. Here, we elucidate lipofuscin composition and report the spatiotemporal dynamics of lipofuscin accumulation in aging and NCL on a neuroanatomical atlas. Multimodal mass spectrometry, ultrastructural analyses, and assays of metabolic flux identify a primary role of the lysosomal-mitochondrial axis in lipofuscin formation. Dissection of implicated molecular pathways reveals protein S-acylation and lipid homeostasis as central processes involved in aging and NCL.

## Main

Lipofuscin is an autofluorescent material that progressively accumulates in the brain and other tissues with age. Although present in many cell types, postmitotic cells are particularly vulnerable to lipofuscin deposition^1^. Lipofuscin was first described by A. Hannover in 1842 as a yellow-brown substance in neurons^2^. Today, it is easily observed by fluorescence microscopy and can be a bothersome background signal or potential confound in immunofluorescence studies of aged tissues. Despite (or perhaps because of) its long history of study and ubiquitous nature, lipofuscin is often dismissed as a fluorescence artifact or an inert wear-and-tear byproduct. As a result, the origin and composition of lipofuscin remain poorly described. An improved biological understanding of this material is needed to assess contributions to aging processes and disease states in which it is observed.

Emerging evidence suggests that lipofuscin can directly disrupt cellular homeostasis and may contribute to neurodegeneration^3^. Indeed, profound lipofuscin accretion occurs in Neuronal Ceroid Lipofuscinoses (NCLs), also known as Batten Disease, a group (*CLN1-CLN14*) of rare monogenic neurodegenerative lysosomal storage disorders with primarily pediatric onset^4^. Lipofuscin (also referred to as ceroid) is the primary neuropathological hallmark of NCLs, which suggests a key etiological role and provides a critical opportunity to investigate the localization, composition, and biogenesis of this material. To dissect the convergent and distinct molecular mechanisms that contribute to lipofuscin formation in aging and NCL, we examined naturally aging wild type (WT) mice and a mouse model of infantile NCL (*CLN1*). *CLN1* is caused by loss-of-function mutations in the gene encoding the enzyme palmitoyl protein thioesterase 1 (*PPT1*)^5,6^. PPT1 catalyzes the removal of S-acyl groups (often palmitic acid) from cysteines on protein substrates^7^, participating in a cycle of reversible post-translational lipidation. S-acylation is increasingly recognized to regulate synaptic and mitochondrial proteins and to play vital roles in neurological function^8^.

Here, we address long-standing questions regarding lipofuscin and identify convergences between aging and NCL that support a mitochondrial/lysosomal axis of aging. Our study details the temporal and neuroanatomical deposition of lipofuscin with histological mapping onto a brain atlas, its organellar origin with ultrastructural analysis, and its protein and lipid composition with a multimodal mass spectrometric approach.

### Development of a fine neuroanatomical atlas of lipofuscin accumulation with aging and *CLN1* progression

**Figure 1.**
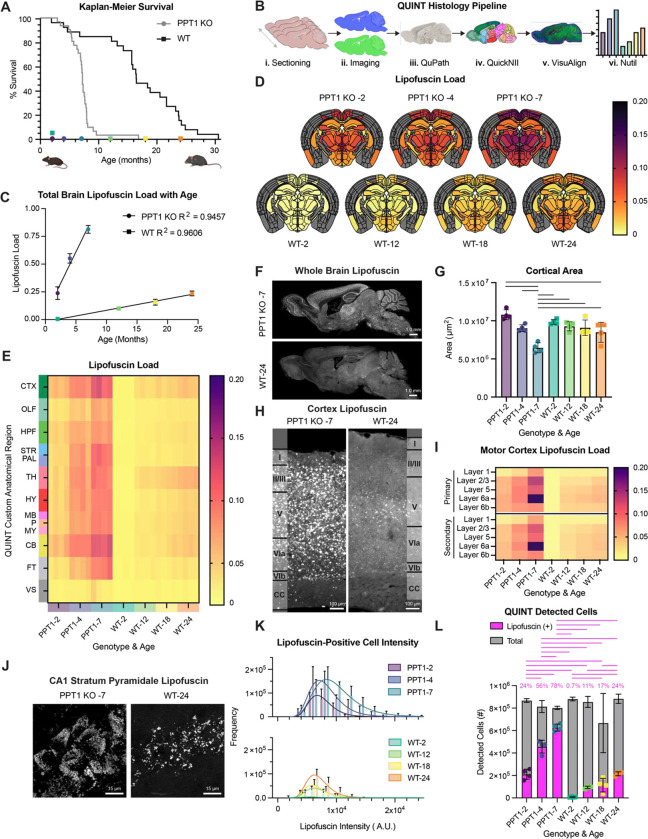
Neuroanatomical atlas of lipofuscin accumulation with age and *CLN1* progression highlights regional vulnerability (**A**) Kaplan-Meier survival of WT (*n =* 26) and PPT1 KO mice (*n =* 31). *p* < 0.001, Mantel-Cox log-rank test. Experimental timepoints as circles (PPT1 KO: 2-, 4-, 7-months) and squares (WT: 2-, 12-, 18-, 24-months). (**B**) Scheme of QUINT histology pipeline. (**C**) Simple linear regression of total brain lipofuscin load with age and *CLN1* progression (WT: y = 0.01025*X − 0.01841; R^2^ = 0.9606; PPT1 KO: y = 0.1129*X + 0.04549; R^2^ = 0.9457). (**D**) Coronal heat maps of average lipofuscin load in medial QUINT reference regions. (**E**) Heat map of lipofuscin load across gross anatomical regions in Allen Mouse Brain Atlas. CTX: isocortex; OLF: olfactory areas; HPF: hippocampal formation; STR: striatum; PAL: pallidum; TH: thalamus; HY: hypothalamus; MB: midbrain; P: pons; MY: medulla; CB: cerebellum; FT: fiber tracts; VS: ventricular system. (**F**) Representative whole brain sagittal sections illustrating autofluorescent storage accumulation at 7 (PPT1 KO) and 24 months (WT) (scale bars, 1.0 mm). (**G**) Quantification of cortical area (*μ*m^2^) with age and *CLN1* progression. (**H**) Representative images of lipofuscin accumulation across motor cortex layers in PPT1-KO 7-month (left) and WT 24-month (right) mice (CC: corpus callosum; scale bar = 100 *μ*m). (**I**) Heat map of average lipofuscin load in primary (top) and secondary (bottom) motor areas by cortical layer. (**J**) Representative high-magnification images illustrating autofluorescent lipofuscin granules in neuronal cell bodies of hippocampal CA1 *stratum pyramidale* in PPT1 KO 7-month (left) and WT 24-month (right) mice (scale bars, 15 *μ*m). (**K**) Histogram of average autofluorescence intensities of lipofuscin-positive cells detected by QUINT. Nonlinear regression envelopes (R^2^: PPT1–2 = 0.8235; PPT1–4 = 0.8265; PPT1–7 = 0.8847; WT-2 = 0.8651; WT-12 = 0.9300; WT-18 = 0.8038; WT-24 = 0.9105). (**L**) Proportion of lipofuscin-positive cells versus total cells detected by QUINT. Graphical data are mean ± SD (*n =* 4 biological replicates/condition; average of *n =* 4 alternate sections/replicate, except K and L, where alternate section data is concatenated for each biological replicate). (G and L) Horizontal lines, *p* < 0.05 by one-way ANOVA with Tukey’s multiple comparisons test. (G) WT cortical atrophy from 2 to 24 months, *p* < 0.05 by *post hoc* student’s t-test. See also **Figure S1**.

To query the accumulation dynamics and neuroanatomical distribution of lipofuscin with aging and *CLN1* progression, we examined brains of wild type (WT; C57BL6/J) and *Ppt1* knockout (PPT1 KO; CgC57BL6/J-*Ppt1*^*tm1Hof*^/J) mice. PPT1 KO mice exhibit significantly shortened lifespan compared to WT (Fig. 1A; underlying graphical data in **Data S1,** throughout)^9,10^. Experimental timepoints were therefore selected to capture median survival of each genotype and ages with robust lipofuscin accumulation based on pilot observations (Fig. 1A; PPT1 KO-2, −4, −7 months; WT-12, −18, −24 months). Two-month-old WT mice (WT-2) were included as a directly comparable experimental timepoint to PPT1 KO and a putative lipofuscin-negative condition. To generate a neuroanatomical atlas of lipofuscin distribution with time, brain sections were subjected to a modified QUINT histology pipeline to segment and quantify lipofuscin signal according to the Allen Brain Atlas (Fig. 1B). Cell detections were determined by DAPI counterstain (Fig. 1Bii), as neurons and glia both accumulate lipofuscin^11^. The resulting histological study is the first anatomical atlas of lipofuscin load with natural aging and with *CLN1* progression in murine brain.

At the whole brain level, lipofuscin load linearly correlated with aging in both WT (y = 0.01025*X Ȓ 0.01841; R^2^ = 0.9606) and PPT1 KO mice (y = 0.1129*X + 0.04549; R^2^ = 0.9457) (Fig. 1C). These data corroborate past studies of WT murine brain^12^ and underscore the utility of lipofuscin load as a ubiquitous aging biomarker, as linear lipofuscin deposition with age can also be observed in humans^13^ and across phyla. Remarkably, lipofuscin accumulated in PPT1 KO mice at 11 times the WT rate, and we did not identify ages within our experimental scheme at which lipofuscin loads were comparable (Fig. 1C).

Lipofuscin load was quantified across 425 fine (Fig. 1D and **Fig. S1A**) and 13 gross neuroanatomical regions defined by the Allen Mouse Brain Atlas (Fig. 1E). The resulting brain atlas revealed specific regional vulnerabilities to autofluorescent storage with patterns of deposition stereotyped for brain region, age, and genotype (**Fig. S1A**). At observation endpoints, lipofuscin puncta were present across most brain areas, with robust accumulation in PPT1 KO (Fig. 1F). In both genotypes, the most affected gross regions were cortex, thalamus, and cerebellum (Fig. 1E). The consistently least affected regions were the ventricular system and fiber tracts, highlighting the primarily somatic localization of lipofuscin (Fig. 1E). Most severely affected fine regions in 7-month PPT1 KO included the nucleus accumbens and numerous cortical areas, while 24-month WT animals displayed the highest lipofuscin loads in subregions of the hypothalamic medial zone (MEZ) and periventricular region (PVR) (**Fig. S1A**). No significant recovery from lipofuscin accumulation was observed with age in any region (**Fig. S1B** and **Fig. S1C**), indicating that lipofuscin biogenesis outpaces its degradation across the brain.

The lipofuscin atlas is a valuable resource to understand regional vulnerability to age. These data also have far-reaching implications for fluorescence microscopy studies of aged mice, in which lipofuscin can be a critical confounding variable. To facilitate use of this temporal atlas, we developed a web tool which allows examination of lipofuscin accumulation in specific fine anatomical regions (https://lipofuscinatlas.yale.edu).

### Salient features of the cortical lipofuscin atlas in *CLN1* and normal aging

Given that cortical atrophy is a prominent feature of NCL/*CLN1*^14,15^ and can be observed in aging^16^, we used the QUINT pipeline to quantitatively capture progressive cortical area changes with age and disease stage (Fig. 1G). PPT1 KO mice exhibited progressive decline in total cortical area, while WT animals displayed a modest decrease in cortical area from 2 to 24 months (Fig. 1G). *CLN1* cortical atrophy was paired with a progressive enlargement in ventricular system area (**Fig. S1D**).

Confocal fluorescence microscopy revealed lipofuscin accumulation in PPT1 KO and aged WT cortices with layer-specific deposition (Fig. 1H), a pattern which was stereotyped across cortical areas, including motor (Fig. 1I) and retrosplenial cortices (**Fig. S1E**). Layer 2/3 and 6a were severely affected in PPT1 KO, and deep cortical layers, particularly layers 5 and 6a, preferentially accumulated lipofuscin in WT (Fig. 1I and **Fig. S1E**).

### Cells with lipofuscin increase in number and fluorescence intensity with age

We next investigated the subcellular distribution of lipofuscin in the hippocampus, as cell body and synaptic layers are easily discernable. In PPT1 KO mice, the *stratum pyramidale* (cell body layer) of the hippocampus has striking lipofuscin pathology, with low loads in neighboring dendritic layers (**Fig. S1F**). Lipofuscin accumulation is also observed in *stratum pyramidale* in aged WT mice, particularly within field CA3 (**Fig. S1F**), although the QUINT pipeline averages across field CA1, CA2, and CA3 (as seen in Fig. 1D) in corresponding quantifications of hippocampal lipofuscin loads (**Fig. S1G**). A similar distribution of lipofuscin autofluorescence is also observed in Purkinje cell bodies in the granule layer of cerebellum (Fig. 1F). Collectively, observations of anatomical areas that contain mostly neuronal cell bodies (**Fig. S1H**) indicate that lipofuscin is primarily concentrated in the soma^17^. Non-neuronal cells were also lipofuscin-positive (**Fig. S1H**), consistent with the observation of lipofuscin in glia^11^.

At high magnification, lipofuscin granules in hippocampal *stratum pyramidale* are observed to progressively fill the cytosol of pyramidal neurons, with a sparser distribution in WT than PPT1 KO (Fig. 1J and **S1I**). Large autofluorescent puncta, such as those shown in PPT1 KO hippocampus (**Fig. S1F**), were thus deconvoluted into numerous lipofuscin granules under high magnification (Fig. 1J and **S1I**). Congruently, the median autofluorescence intensity per lipofuscin-positive cell increased with both aging and *CLN1* progression (Fig. 1K), as did the proportion of lipofuscin-positive cells in total brain (Fig. 1L). These data indicate that increases in lipofuscin load are caused by 1) *de novo* lipofuscin formation or uptake in previously unaffected cells, and 2) by an increase in the number and/or brightness of lipofuscin granules within individual cells. Lipofuscin granules in mouse brain appeared similar morphologically to those found in human dorsolateral prefrontal cortex in aged healthy controls (**Fig. S1J**).

### Purification of autofluorescent lipofuscin granules by brain fractionation

**Figure 2.**
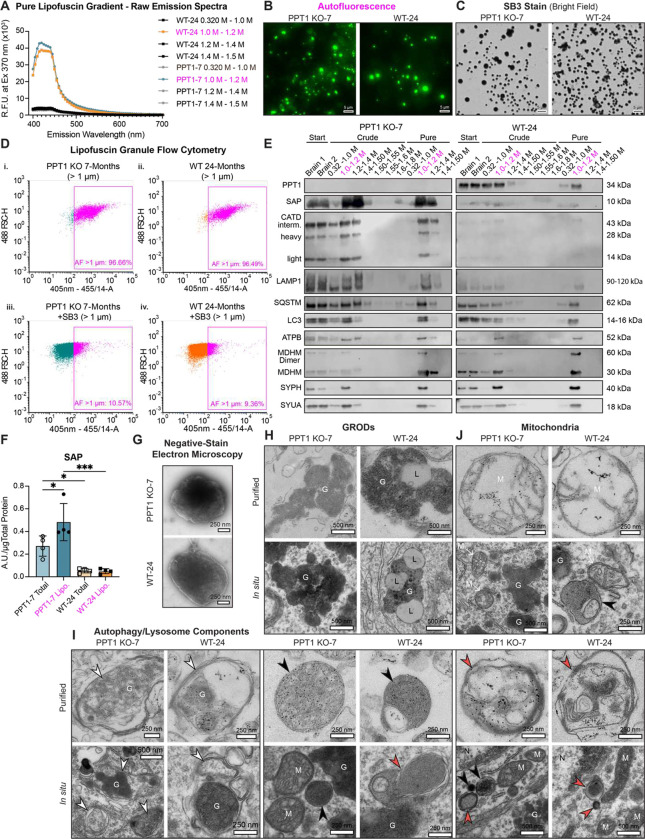
Purification of autofluorescent lipofuscin granules by brain fractionation (**A**) Representative autofluorescence emission spectra of brain fractions following discontinuous sucrose gradient ultracentrifugation. Data are mean ± SD (*n =* 3 technical replicates). (**B** and **C**) Representative images of lipofuscin granules taken with (B) native fluorescence microscopy or (C) bright field microscopy following solvent black 3 (SB3) staining (scale bars, 5 *μ*m). (**D**) Flow cytometry of autofluorescent granules (> 1 *μ*m in diameter (*μ*m)) before (**i** and **iii**) and after (**ii** and **iv**) quenching with solvent black 3 (SB3) (>50,000 events per sample run). Percentages (%) in purple gate indicate proportion of autofluorescent particles detected above autofluorescence threshold. (**E**) Quantitative immunoblotting of total brain homogenates (Start), crude lipofuscin gradient fractions (Crude), and pure lipofuscin gradient fractions (Pure) for markers of *CLN1* lipofuscin (SAP), lysosomal hydrolases (PPT1, CATD: interm. = intermediate, heavy-chain, and light-chain), lysosomal markers (LAMP1), autophagy markers (LC3, SQSTM), mitochondrial proteins (ATPB, MDHM) and synaptic proteins (SYUA, SYPH). Purple, autofluorescent fractions. (**F**) Quantification of SAP protein enrichment by immunoblotting of total brain and purified lipofuscin. * *p* < 0.05; *** *p* < 0.001, one-way ANOVA with Tukey’s multiple comparisons test. (**G**) Representative negative-stain electron micrographs of purified autofluorescent granules (scale bars, 250 nm). (**H-J**) Representative electron micrographs of (H) granular osmiophilic deposit (GROD) structures with associated lipid droplets, (I) putative late endosomes/autolysosomes (white arrowheads), dense core lysosomes (black arrowheads), and multilamellar lysosomes (red arrowheads), and (J) mitochondria with disordered, sparse, or degraded cristae in autofluorescent fractions purified from brain (top) and *in situ* in brain sections (bottom) (scale bars, 250 or 500 nm; G, GROD; L, lipid droplet; M, mitochondria). Samples derived from PPT1 KO 7-month and WT 24-month brain, throughout. See also **Figures S2**, **S3**, **S4**, and **S5**.

To analyze the organellar biogenesis and molecular composition of lipofuscin in *CLN1* and aging, we purified autofluorescent granules from murine brain with established techniques^18–21^ modified to improve gradient separation (see Methods). Subcellular fractionation of WT and PPT1 KO brain tissues resulted in a visually yellow-brown fraction that typically sedimented at the 1.0–1.2 M sucrose interface (**Fig. S2A**). This brain fraction was strongly autofluorescent compared to others by fluorescence spectroscopy (Fig. 2A). Excitation at 370 nm produced emission spectra characteristic of lipofuscin^18,22^, with a broad emission peak at 425–440 nm (Fig. 2A). Purified granules remained autofluorescent following freeze-thaw cycle and storage at 4°C (Fig. 2B). Autofluorescence was quenched by ready uptake of the lipophilic dye solvent black 3 (SB3) (Fig. 2C and 2D), a classic lipofuscin stain^23^. Flow cytometry sorting of quenched and unquenched granules from each genotype (>1 *μ*m in diameter) demonstrated that over 86% of isolated granules are autofluorescent (Fig. 2D and **Fig. S2B**), highlighting preparation purity. Bright field imaging of SB3-stained granules revealed consistently circular shape (**Fig. S2C**). Average granule diameter across ages and genotypes was observed to be 1.38 *μ*m (**Fig. S2D**), consistent with prior morphological descriptions^17^ and observations of granule size obtained by flow cytometry (**Fig. S2B**).

Pure lipofuscin isolated from WT mice contained PPT1, while this thioesterase was absent in KO fractions (Fig. 2E). Compared to WT and total brain, PPT1 KO lipofuscin was significantly enriched for saposin (SAP) (Fig. 2F), the primary known protein component of *CLN1* lipofuscin^24^. PPT1 KO lipofuscin was also enriched for cathepsin D (CATD) (Fig. 2E), a lysosomal hydrolase and the causal gene for *CLN10*, known to be persistently upregulated in brain and spinal cord in *CLN1*^*7,*25*,*26^. Across genotypes, pure lipofuscin contained established lysosome (LAMP1), autophagy (LC3, SQSTM (p62)), and synapse markers (SYUA, SYPH) (Fig. 2E). Consistent with immunoblots, CATD and LAMP1 expression were significantly upregulated in PPT1 KO hippocampus compared to WT, where these markers colocalized with lipofuscin (**Fig. S3A-D**). Increased LAMP1 and CATD expression was also observed in primary cortical cultures in the absence of autofluorescence at DIV14 (**Fig. S3E-G**). Mitochondrial markers (ATPB, MDHM) were also observed in purified lipofuscin (Fig. 2E). Most mitochondria are removed early in the lipofuscin purification process, and residual mitochondria are separated from the autofluorescent fraction by sedimentation to the 1.50–1.55 M sucrose interface, and consecutively by pelleting (**Fig. S2A**). Thus, the presence of mitochondrial proteins in the autofluorescent fraction suggests that lipofuscin contains rarer dysfunctional mitochondria or digested mitochondrial components, consistent with theories that posit lipofuscin forms from insufficient lysosomal degradation of oxidatively damaging mitochondria^27–30^.

### Autofluorescent granules include granular osmiophilic deposits (GRODs), autophagy-lysosome components, and damaged mitochondria

Few ultrastructural studies of *CLN1* lipofuscin have been conducted in brain tissue^24,31^, and morphology of lipofuscin inclusions across aging and disease states is heterogenous^14,32–37^. To identify and compare the ultrastructure of purified and *in situ* lipofuscin in advanced *CLN1* and aged WT mouse brain, we subjected purified autofluorescent fractions and brain sections to electron microscopy (EM). By negative-stain EM, purified granules are electron-dense, membrane-bound structures (Fig. 2G), validating the lipid-rich character of lipofuscin indicated by bulk measurements of its composition^38^. Brain sections revealed deposition of electron-dense membrane-delimited granular structures in PPT1 KO 7-month-old and WT 24-month-old motor cortex and CA3 of hippocampus (Fig. 2H and **Fig. S4A**). Similar structures, amongst others, were also observed in purified autofluorescent fractions (Fig. 2H and **Fig. S4A**). These electron-dense components are consistent with GRODs observed in *CLN1* patient-derived tissues and cells^24,31,35^, and contrast with curvilinear structures found in other NCL subtypes^33,39–42^. WT structures (Fig. 2H and **Fig. S4A**) also corroborate prior observations of the WT aging brain, in which osmiophilic deposits may associate with lipid droplets, appear GROD-like, or sometimes contain lamellar structures^34,37^.

Across all measured cells, fewer GRODs were detected in WT than in PPT1 KO brains (**Fig. S4B**). These osmiophilic structures occupied an average of 6.2% (WT) and 11.0% (PPT1 KO) of the cytosolic area (**Fig. S4C**). This modest fold-change, compared to the >3-fold increase in lipofuscin load by autofluorescence (Fig. 1A and Fig. 1E), suggests the contribution of additional organellar structures to autofluorescent storage. Strikingly, while WT GRODs were often associated with lipid droplets, lipid droplets were not observed in *CLN1* brains by EM (**Fig. S4D**). These data indicate a fundamental difference in the ultrastructure of canonical lipofuscin in each genotype.

Secondary lysosomes with electron-dense inclusions are also considered lipofuscin, as they likely represent an earlier phase of lipofuscinogenesis than terminal GRODs. We observed (Fig. 2I and **Fig. S4E**) putative late endosomes or autolysosomes, sometimes containing GRODs (G), granular dense core lysosomes (black arrowheads), and/or multilamellar lysosomes (red arrowheads)^43–46^ in both genotypes *in situ* and in purified autofluorescent fractions. *In situ*, lysosomes occasionally contacted GROD structures or mitochondria (Fig. 2I and **Fig. S4E**). Mitochondria with somewhat disordered cristae were also observed in the soma (Fig. 2J) and near synaptic densities (**Fig. S4F**). In purified autofluorescent fractions, mitochondria with severely degraded cristae were observed, occasionally within multilamellar structures, (Fig. 2J and **Fig. S4F**), a phenotype likely to have been exacerbated by the purification process. There were no differences between genotypes in the average number of mitochondria per soma in observed brain regions (**Fig. S4G**). However, we did note a minor increase in mitochondrial area in PPT1 KO brains (**Fig. S4H**), and a shift from a tubular mitochondrial morphology in WT to a rounder morphology in PPT1 KO (**Fig. S4I**), indicative of possible mitochondrial dysfunction^47–49^.

Together, these data strongly support a lysosomal contribution to lipofuscin biogenesis^1,38^, and are consistent with the established hypothesis that lipofuscin contains oxidatively damaged or dysfunctional mitochondrial components^27,29,30^. Taken together with flow cytometry findings which demonstrate the fluorescence of most purified granules (Fig. 2D and **Fig. S2B**), these data implicate components present in both GRODs and multiple organellar structures as sources of autofluorescence.

### Loss of PPT1 function causes mitochondrial deficits independent of lipofuscin formation

Since we observed mitochondrial structures in purified autofluorescent fractions (Fig. 2J) and a difference in mitochondrial morphology between genotypes (**Fig. S4H** and **Fig. S4I**), we directly queried metabolic flux in live CRISPR-engineered PPT1 KO HEK293T cells (**Fig S5A**). HEK293T cells allowed us to test mitochondrial function in the absence of lipofuscin, as these cells frequently divide and thus do not accumulate autofluorescent material. PPT1 KO cells were metabolically dysfunctional compared to WT controls (**Fig. S5B**). Specifically, PPT1 KO cells had decreased basal respiration (**Fig. S5B** and **Fig. S5C**). ATP production was also decreased (**Fig. S5D**), which implicates diminished substrate availability or disruption of the electron transport chain. Coupling efficiency, the proportion of oxygen consumption related to ATP synthesis, was also reduced in PPT1 KO cells (**Fig. S5E**). PPT1 deficiency also induced an increase in extracellular acidification rate (ECAR) (**Fig. S5F**), indicating a possible compensatory upregulation of glycolytic activity. Finally, PPT1 KO cells exhibited unchanged proton leak and maximal respiration (**Fig. S5G** and **Fig. S5H**) and increased spare respiratory capacity (**Fig. S5I**), indicating that mitochondria may retain adaptability to meet energy demands despite the baseline reductions in respiration and ATP production. Together, these data demonstrate that PPT1 enzyme deficiency is sufficient to cause mitochondrial stress and an increased reliance on glycolysis.

### Elucidation of the lipofuscin proteome in *CLN1* and the WT aging brain reveals enrichment in S-acylated proteins

**Figure 3.**
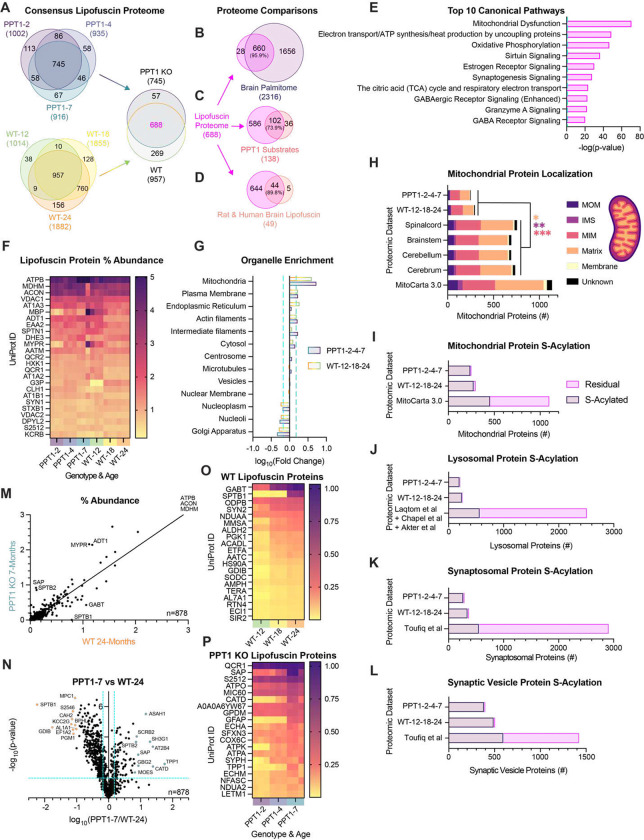
Elucidation of the lipofuscin proteome reveals mitochondrial and lysosomal constituents prone to modification by S-acylation (**A**) Venn diagram illustrating consensus proteins identified in autofluorescent lipofuscin fractions by LFQ-MS across timepoints (months) and genotypes (PPT1–2-4–6: *n =* 745; WT-12–18-24: *n =* 957; consensus lipofuscin proteome: *n =* 688). (**B-D**) Venn diagrams of consensus lipofuscin proteome (*n =* 688) and published proteomes: (B) whole brain and synaptosomes following Acyl Resin-Assisted Capture of S-acylated proteins^7^ (660/688; 95.9%), (C) known PPT1 substrates (*n =* 138)^7^ (102/138; 73.9%), (D) known rat and human brain lipofuscin proteins (*n =* 49)^18^ (44/49; 89.8%). (**E**) Top 10 canonical pathways by Ingenuity Pathway Analysis of consensus lipofuscin proteome hits (*n =* 688). (**F**) Percent (%) abundance heatmap of top 25 most abundant lipofuscin proteins (UniProt ID; scale, 0.15 – 5%; values > 5%, dark purple for illustrative purposes). (**G**) Annotation and fold-change enrichment of consensus lipofuscin proteomes (PPT1–2-4–7: *n =* 745; WT-12–18-24: *n =* 957) by primary localization in Human Protein Atlas^55^. (**H**) Annotation of mitochondrial proteins in consensus lipofuscin proteomes (PPT1 KO: *n =* 250/745; WT: *n =* 296/957) and mitochondrial isolates from different brain regions by sub-organellar localization in MitoCarta 3.0^56^. MOM: mitochondrial outer membrane; IMS: intermembrane space; MIM: mitochondrial inner membrane. * *p* < 0.05, ** *p* < 0.01, *** *p* < 0.001, unpaired two-tailed t-test. (**I-L**) Overlap of consensus lipofuscin proteomes and annotation datasets by organelle with presence in S-acylated brain proteome^7^: (I) mitochondrial proteins (PPT1 KO: *n =* 250; WT: *n =* 296; MitoCarta 3.0^56^: *n =* 1099), (J) lysosomal proteins (PPT1 KO: *n =* 205; WT: *n =* 249; combined lysosomal annotation datasets^57–59^: *n =* 2517), (K) synaptic proteins (PPT1 KO: *n =* 285; WT: *n =* 369; brain-derived synaptosome fraction dataset^60^: *n =* 2921), (L) synaptic vesicle proteins (PPT1 KO: *n =* 401; WT: *n =* 509; brain-derived purified synaptic vesicle dataset^60^: *n =* 1426). (**M**) Correlation of percent (%) abundance between WT 24-month and PPT1 KO 7-month lipofuscin proteomes (*n =* 878 overlapping proteins; R^2^ = 0.8983; y = 1.035*X −0.008143). For illustrative purposes, ATPB, ACON, and MDHM fall outside axis limit. (**N**) Fold-change comparison between WT 24-month and PPT1 KO 7-month lipofuscin proteomes (*n =* 878). (**O-P**) Heatmaps of percent (%) abundance of top 25 proteins identified in (O) all WT timepoints, or (P) all PPT1 KO timepoints with significant progressive accumulation in lipofuscin (*p* < 0.05, unpaired two-tailed t-test). Proteomic data represent *n =* 3 sex-matched biological replicates; *n =* 3 technical replicate injections. Blue dashed lines, significant thresholds at 1.5-fold-change or *p* = 0.05. See also **Figures S6** and **S7**.

Few proteomic studies have been conducted on brain-derived lipofuscin^18,50^, as most have focused on retinal storage material^21,22,51^, which highlights a need to probe lipofuscin protein content with modern mass spectrometry tools. To elucidate convergent composition and unique protein signatures of autofluorescent storage material isolated from WT and *CLN1* mouse brains, we performed label-free quantification mass spectrometry (LFQ-MS) with aging and disease progression. The result is the largest lipofuscin proteome to date, which details a core protein set across timepoints for each genotype (PPT1 KO: *n =* 745; WT: *n =* 957). Strikingly, many lipofuscin proteins were shared in a consensus set between genotypes and across timepoints (*n =* 688) (Fig. 3A).

Protein constituents of *CLN1* lipofuscin have long been hypothesized to be undigested, palmitoylated substrates of PPT1^52^. To determine whether lipofuscin proteins are palmitoylated (or S-acylated) under homeostatic conditions, we compared the core lipofuscin proteome (*n =* 688) to our published dataset of S-acylated proteins isolated from mouse brain by acyl-resin assisted capture (*n =* 2316)^7^. Remarkably, nearly all detected lipofuscin proteins (95.6%) can be S-acylated (Fig. 3B). This finding constitutes a substantial enrichment above chance, as approximately 20% of proteins are estimated to be modified by S-acylation under homeostatic conditions^53,54^. We also compared the consensus lipofuscin proteome to our list of validated PPT1 substrates (*n =* 138)^7^ and identified the majority to be present (*n =* 102; 73.9%) (Fig. 3C). Since PPT1 substrate identities were hitherto unknown, these data provide primary evidence for the seminal hypothesis that PPT1 substrates accumulate in lipofuscin^52^. Together, these data suggest that brain aging and *CLN1* are functionally related by dysregulation of S-acylated proteins. Comparison of the consensus lipofuscin proteome to the single proteome of purified lipofuscin in the literature^18^ (*n =* 49 proteins) also revealed a high degree of overlap (*n =* 44; 88.9%; Fig. 3D), corroborating our purification method and highlighting the translational applicability of our findings^18^ (**Data S2**).

Examination of the core lipofuscin proteome (*n =* 688) by ingenuity pathway analysis (IPA) implicated mitochondrial dysfunction and mitochondrial respiration (Fig. 3E), in alignment with a mitochondrial and a reactive oxygen species (ROS) contribution to lipofuscin biogenesis^27^. Independent pathway analysis of lipofuscin proteins for each genotype (Fig. 3A) also revealed common involvement of mitochondrial protein degradation and Parkinson’s Disease signaling (**Fig. S6A** and **Fig. S6B**) (**Data S3**). Several mitochondrial proteins were the most abundant lipofuscin constituents across all conditions (Fig. 3F). The beta subunit of the mitochondrial ATP synthase (ATPB), the mitochondrial enzymes malate dehydrogenase (MDHM) and aconitase (ACON), and the critical channel VDAC1 each comprised a notable 2–5% of total lipofuscin protein (Fig. 3F). Each of these proteins were detected in our dataset of S-acylated proteins^7^. Amongst this set, S-acylated cysteines were confirmed for MDHM, ACON, and VDAC1, of which ACON was also classified as a PPT1 substrate^7^ (**Data S2**).

To confirm which organellar components were enriched in the lipofuscin proteome in an unbiased manner, we annotated orthologs by subcellular localization using the Human Protein Atlas (www.proteinatlas.org)^55^. Mitochondrial and plasma membrane components were significantly enriched in lipofuscin, while nucleoplasm, nucleoli, and Golgi apparatus proteins were significantly de-enriched (Fig. 3G). PPT1 KO and WT lipofuscin proteins were also compared to proteomes of mitochondrial isolates derived from various mouse brain tissues^56^, revealing a subset (PPT1 KO: *n =* 250; WT: *n =* 296) of mitochondrial proteins to be present (Fig. 3H). Examination of sub-organellar compartment localization indicated a higher ratio of mitochondrial inner membrane (MIM) proteins and a reduced ratio of matrix and intermembrane space proteins (IMS) in lipofuscin compared to mitochondrial isolates (Fig. 3H) (**Data S2**). These data suggest that membrane-bound mitochondrial proteins are more likely to persist in lipofuscin than soluble proteins and reinforce that lipofuscin is devoid of intact mitochondrial contaminants.

Autophagy-lysosome structures were observed in autofluorescent fractions by EM (Fig. 2I and **Fig. S4E**), but the Human Protein Atlas contained few lysosomal proteins (*n =* 19)^55^. To adequately assess lysosomal enrichment, we performed annotation of lipofuscin proteins for lysosomal localization according to established annotations and proteomic analyses of lysosomal isolates^57^ and lysosome-enriched fractions^58,59^. Similarly, to adequately assess enrichment of synapse-resident proteins, many of which are S-acylated^7^, we compared lipofuscin proteins to established annotations of synaptosomal (Fig. 3K) and synaptic vesicle (Fig. 3L) brain isolates^60^. In alignment with the consensus lipofuscin proteome (Fig. 3B) we found most (>94%) lipofuscin proteins with mitochondrial (Fig. 3I), lysosomal (Fig. 3J), synaptosomal (Fig. 3K), or synaptic vesicle (Fig. 3L) annotation to be capable of S-acylation ^7^. In contrast, the remaining proteins from each annotation dataset that were not detected in lipofuscin contained a much smaller proportion of proteins (<17%) that overlapped with our S-acylated proteome^7^ (Fig. 3I–L) (**Data S2**). Together, these data suggest that S-acylated mitochondrial, synaptic, and lysosomal proteins are uniquely disposed to be present in autofluorescent lipofuscin structures.

### WT lipofuscin implicates a redox response while Lysosomal Storage Disorder enzymes define *CLN1* lipofuscin

Comparison of late-stage lipofuscin proteomes (WT-24 and PPT1–7) revealed a large intersection in protein constituents (*n =* 878) and a strong correlation between percent abundances (R^2^ = 0.8983; m = 1.035) (Fig. 3M). Significantly enriched proteins in each genotype (Fig. 3N) point to common mitochondrial dysfunction, but other affected pathways diverge (**Fig. S6C** and **S6D**). PPT1 KO 7-month lipofuscin implicates sirtuin, neutrophil, and Huntington’s Disease signaling pathways (**Fig. S6C**). In contrast, WT 24-month lipofuscin is enriched in proteins involved in synaptogenesis, clathrin mediated-endocytosis, and Parkinson’s Disease signaling (**Fig. S6D**) (**Data S3**).

We also examined consensus proteins for each genotype (Fig. 3A) that underwent significant progressive accumulation in lipofuscin (PPT1: *n =* 53; WT: *n =* 66). Amongst this subset, diverse mitochondrial enzymes progressively accumulated in lipofuscin in both genotypes (Fig. 3O and Fig. 3P). In WT lipofuscin, several heat shock proteins (HS90A, TRAP1, HS105), additional chaperones (TCPH, BCS1), and oxioreductase proteins (SODC, ALDH2), accumulated with age (Fig. 3O). Instead of proteins with chaperone or redox function, PPT1 KO lipofuscin exhibited accretion of several proteins implicated in NCLs and other lysosomal storage disorders (SAP, CATD, TPP1, SCRB2, ASAH1) (Fig. 3P). Several of these proteins were also amongst the highest differentially abundant in PPT1 KO lipofuscin compared to WT (Fig. 3N). The progressive accumulation and high abundance of prosaposin (SAP) in lipofuscin corroborate use of this protein as a *CLN1* biomarker^24^, but emphasize other possible biomarker proteins (Fig. 3N and Fig. 3P). As the respective causal genes for *CLN2* and *CLN10*, the accumulation of the lysosomal proteases tripeptidyl peptidase 1 (TPP1) and cathepsin D (CATD), support emerging molecular networking amongst NCL disorders^61–65^. Similarly, the accumulation of the glucocerebrosidase (GBA1) receptor SCRB2, a myoclonic epilepsy factor^66^ and modifier of Gaucher Disease severity^67^, and the Farber Disease causal gene product ASAH1^68^, indicate a possible etiological axis linking lysosomal storage disorders.

### *CLN1* and aging involve dysregulation of multiple lysosomal enzymes

To query the mechanistic contribution of lysosomal enzymes to *CLN1-* and age-related lipofuscin, we examined levels and enzymatic activity in whole brain and purified lipofuscin. CATD protein was significantly elevated with *CLN1* progression and compared to WT (**Fig. S7A** and **Fig. S7B**). CATD enzyme activity followed these trends at the total brain level (**Fig. S7C**). However, specific CATD activity (normalized to CATD protein levels) fell below WT levels in *CLN1* brain and declined with *CLN1* progression (**Fig. S7D**). Given the high levels of pro-forms of CATD in PPT1 KO brains (**Fig. S7A**), CATD maturation may be inhibited. TPP1 exhibited a progressive increase in activity in both WT and *CLN1* brains with significantly higher levels in *CLN1* (**Fig. S7E**). TPP1 activity levels were consistent with proteomic data (Fig. 3P). CATD, TPP1, and PPT1 were enzymatically active in lipofuscin (**Fig. S7F**), suggesting that intact autolysosomes in the autofluorescent fraction may be partially functional.

Due to the progressive accumulation of the GBA1 receptor SCRB2 and the GBA1 activator SAP in lipofuscin, we also examined GBA1 activity in total brain homogenates (**Fig. S7G**), as GBA1 was not detected in the lipofuscin proteome. GBA1 activity was deficient at early stage *CLN1*, then recovered by 7-months to WT levels (**Fig. S7G**). We also observed a modest decline in GBA1 activity with WT aging (**Fig. S7G**). Together, these data point to the activation and co-regulation of lysosomal enzymes as a key feature of NCLs and implicate this lysosomal network^62,69^ in brain aging.

### Spatial mass spectrometric lipid correlates of lipofuscin autofluorescence in situ

**Figure 4.**
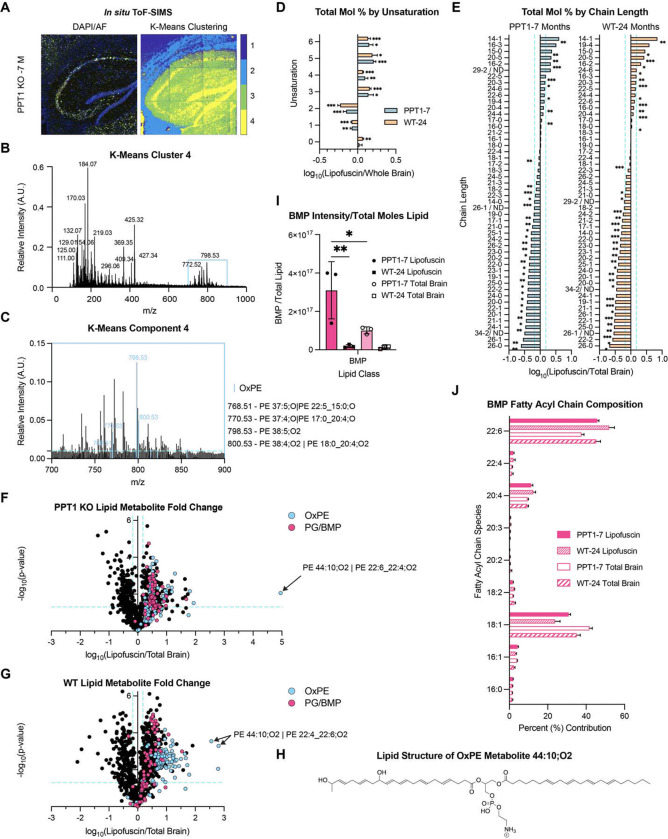
Lipofuscin contains polyunsaturated fatty acids, oxidized lipids, and lipids with mitochondrial and lysosomal origin (**A**) Lipofuscin autofluorescence (AF; yellow) and DAPI counterstain (blue) in hippocampal formation (left), and a serial brain section subjected to ToF-SIMS and K-means clustering analysis of resulting spectral image (right). (**B** and **C**) ToF-SIMS spectrum of K-means cluster 4 (B) with mass-charge (m/z) of major peaks. Inset (C), canonical lipid peaks (m/z 700–900) with putative peak assignments (mass-tolerance, 0.02 Da) for oxidized phosphatidylethanolamine (OxPE) metabolites (blue). (**D-E**) Unbiased lipidomics fold-change comparisons between lipofuscin and total brain lipid mol percent (%) by (D) unsaturation and (E) chain length for PPT1 KO 7-month (blue) and WT 24-month (orange) samples. ND, Not Determined. (**F-G**) Fold-change comparison between lipofuscin and total brain lipid metabolites from (F) PPT1 KO 7-month and (G) WT 24-month animals. OxPE (blue), phosphatidylglycerol/bis(monoacylglycero)phosphate (PG/BMP; pink), all lipid classes (**Fig. S10C** and **Fig. S10D**). (**H**) Putative chemical structure of lipofuscin-enriched OxPE metabolite PE44:10;02. (**I** and **J**) BMP isomer (I) intensity normalized to total lipid, and (J) percent (%) contribution (>1%) of fatty acyl chain species to composition, in PPT1 KO 7-month and WT 24-month lipofuscin and total brain. Lipidomic data are mean ± SD (*n =* 3 biological replicates). Blue dashed lines indicate significance thresholds at 1.5-fold-change or *p* = 0.05. * *p* < 0.05, ** *p* < 0.01, *** *p* < 0.001 by (D-G) unpaired two-tailed t-test or (I) one-way ANOVA with Tukey’s multiple comparison test. See also **Figures S8**, **S9**, and **S10**.

To identify non-protein lipofuscin components in an unbiased manner, we performed time-of-flight secondary ion mass spectrometry (ToF-SIMS) of purified lipofuscin fractions (**Fig. S8**) and *in situ* lipofuscin in mouse brain sections (Fig. 4A). ToF-SIMS spectra of purified lipofuscin revealed consistent ions between genotypes in both negative and positive mode (**Fig. S8A and Fig. S8B**), underscoring the similarity of *CLN1* and age-related lipofuscin. Putative identity assignments for major [M-H]^−^ peaks included characteristic phosphate molecular ion and lipid head group fragments (m/z 78.97, 96.97, 140.03, 153.01) and fatty acyl chains including palmitic acid (16:0; m/z 255.26), oleic acid (18:1; m/z 281.27), stearic acid (18:0; m/z 283.29), arachidonic acid (20:4; m/z 303.25), and docosahexaenoic acid (DHA; 22:6; m/z 327.28)^70^ (**Fig. S8A**).

To identify spatial mass-spectrometric correlates of lipofuscin *in situ*, we performed K-means clustering analysis of ToF-SIMS spectral images of PPT1 KO 7-month brain sections, which have robust lipofuscin deposition (Fig. 4A). The hippocampus was targeted in this analysis due to the spatial segregation of lipofuscin to the *stratum pyramidale* and relative absence in surrounding areas (**Fig. S1F**). This analysis revealed a cluster in the hippocampus (Cluster 4) that aligned with the spatial distribution of lipofuscin autofluorescence in alternate sections (Fig. 4A). The spectrum for this cluster included a series of canonical lipid peaks (m/z 700–900) (Fig. 4B). Comparison of ToF-SIMS peaks in cluster 4 to lipid reference spectra identified several putative overlapping hits corresponding to oxidized phosphatidylethanolamine (OxPE) metabolites (mass tolerance of m/z 0.02) (Fig. 4C).

### Lipofuscin is enriched for long-chain polyunsaturated fatty acids (PUFAs)

To conduct high-throughput identification of lipofuscin lipid components, we performed untargeted lipidomics of lipid extracts of purified lipofuscin and total brain homogenates. Across genotypes, lipofuscin was enriched for polyunsaturated fatty acids (PUFAs), while saturated and monounsaturated fatty acids predominated in total brain (Fig. 4D and Fig. 4E). Significantly enriched lipofuscin PUFAs included the long-chain omega-3 fatty acids docosahexaenoic acid (DHA; 22:6), docosapentaenoic acid (DPA; 22:5), and eicosapentaenoic acid (EPA; 20:5), and the omega-6 arachidonic acid (20:4) (Fig. 4E).

### Lipofuscin lipids corroborate mitochondrial and lysosomal content and implicate lipid oxidation

Lipidomics analysis of lipofuscin and total brain homogenates revealed numerous significant differences in percent composition by lipid class (**Fig. S9A** and **Fig. S9B**). Fold-change comparisons in the abundance of individual lipofuscin lipid metabolites between genotypes revealed striking differential upregulation of many phosphatidylglycerol/bis(monoacylglycerol)phosphate (PG/BMP – structural isomers) species in PPT1 KO (**Fig. S9B** and **Fig. S10A**). Lysolipids, including the BMP precursor lyso-phosphatidylglycerol (LPG), were also upregulated in PPT1 KO lipofuscin, along with oxidized phosphatidylinositol (OxPI) and oxidized phosphatidylethanolamine (OxPE) metabolites (**Fig. S10A**). In contrast, few lipid metabolites were specific for WT lipofuscin compared to PPT1 KO (**Fig. S10A**). These changes in lipofuscin lipid composition between genotypes were also reflected in total brain (**Fig. S10B**).

To determine which specific lipids were enriched in lipofuscin, we next examined fold-change of metabolites between lipofuscin and total brain for each genotype (Fig. 4F and 4G). PPT1 KO lipofuscin was enriched for cardiolipin (CL), LPG, OxPE, and PG/BMP species (Fig. 4F and **Fig. S10C**). WT lipofuscin was also enriched for these species, with the addition of triglyceride (TG), acylated hexosylceramide (AHexCer), lyso-PI (LPI), OxPI, EtherPI, and PI (Fig. 4G and **Fig. S10D**). In comparison to total brain, PPT1 KO lipofuscin was de-enriched for diglyceride (DG), triglyceride (TG), the sphingolipid ceramide (Cer), and the glycosphingolipids hexosylceramide (HexCer) and sulphated hexosyl ceramide (SHexCer) (**Fig. S10C**). WT lipofuscin was also de-enriched for these lipid species, except for triglyceride (**Fig. S10D**).

The majority of OxPE lipid metabolites were enriched in lipofuscin compared to total brain (Fig. 4F and Fig. 4G) and in PPT1 KO compared to WT (**Fig. S10A** and **Fig. S10B**), supporting the spatial colocalization of these components with lipofuscin autofluorescence. The predominance of long-chain polyunsaturated OxPE species in lipofuscin (e.g. PE 38:5;O2 in Fig. 4C and PE 44:10; O2 in Fig. 4F–H) is of particular interest, as the oxidation of these lipids can theoretically produce cyclized and resonant structures that could contribute to lipofuscin autofluorescence. Triglyceride content in WT lipofuscin is consistent with the detection of lipid droplets in WT autofluorescent fractions (Fig. 2H and **Fig. S4A**), which are primarily composed of this lipid^71^. The enrichment of cardiolipin, a lipid derived exclusively from mitochondria^72^, in lipofuscin from both genotypes, corroborates the high mitochondrial content of the lipofuscin proteome (Fig. 3).

**Figure 5.**
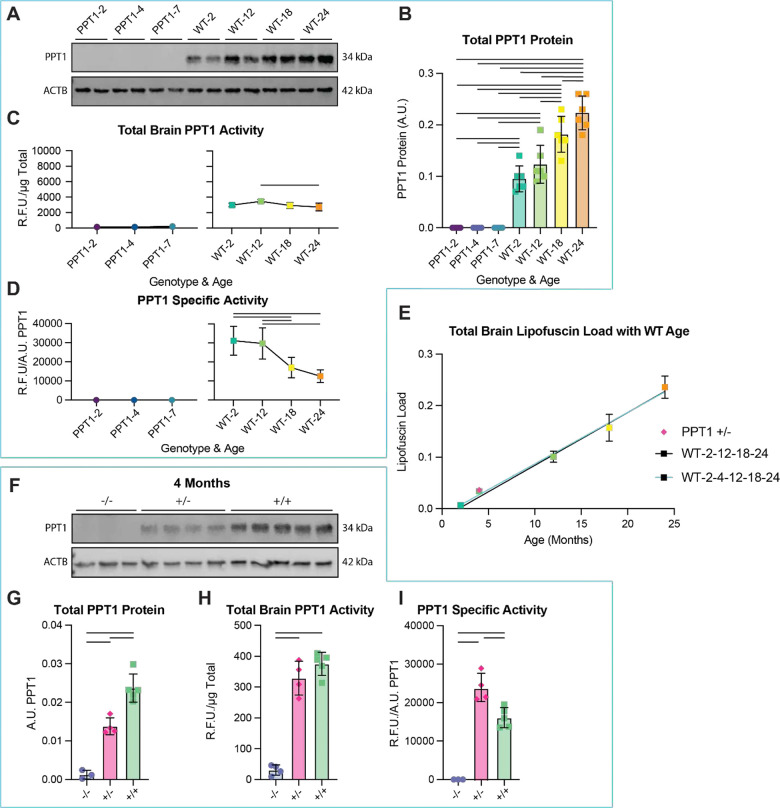
PPT1 specific activity is a predictor of lipofuscin load (**A** and **B**) Representative immunoblot (A) of PPT1 with age and *CLN1* with actin (ACTB) loading control in total brain homogenates and (B) quantification normalized to ACTB. (**C**) Bulk PPT1 enzyme activity (R.F.U.) in total brain fractions normalized to total protein (*μ*g). (**D**) Specific enzyme activity of PPT1 (R.F.U./A.U. PPT1 protein) in total brain homogenates. (**E**) Holdout validation of WT and *Ppt1* +/− 4-month total brain lipofuscin loads with simple linear regression model of WT aging (as in Fig. 1C). Black line, original regression fit (WT: y = 0.01025*X – 0.01841; R^2^ = 0.9606). Blue line, improved simple linear regression fit with WT 4-month timepoint (y = 0.009991*X – 0.01297; R^2^ = 0.9656). (**F** and **G**) Immunoblot of PPT1 (F) in 4-month total brain homogenates (*Ppt1* +/+, +/−, or −/−) with actin (ACTB) loading control, and (G) quantification normalized to ACTB. (**H**) Bulk PPT1 enzyme activity (R.F.U.) in 4-month-old total brain fractions (*Ppt1* +/+, +/−, or −/−) normalized to total protein (*μ*g). (**I**) Specific enzyme activity of PPT1 in 4-month-old animals (*Ppt1* +/+, +/−, or −/−) (R.F.U./A.U. PPT1 protein). Data are mean ± SD ((A-B) *n =* 5–6, (C-D) *n =* 4–5, (E-I) *n =* 3–5 biological replicates). Horizontal bars, *p* < 0.05 by two- (B) or one-way (C-E, G-I) ANOVA with Tukey’s multiple comparisons test.

As lipidomics analysis was conducted in negative ion mode, we could not initially detect PG and BMP metabolites as discrete lipid classes^73^. To distinguish between PG and BMP isomers^74^, we examined BMP-specific ammonium adducts in positive mode, which revealed a 3-fold enrichment of BMP in PPT1 KO lipofuscin compared to total brain, and a 20-fold enrichment in PPT1 KO vs. WT lipofuscin (Fig. 4I). We next examined the contribution of fatty acyl groups to the BMP (Fig. 4J) and PG/BMP signals (**Fig. S8E**). DHA (22:6) was the predominant BMP species (Fig. 5J), likely contributing a substantial portion to the increased PG/BMP 22:6 signal in PPT1 KO over WT (**Fig. S10E**). BMP also contained high proportions of oleic acid (18:1) and arachidonic acid (20:4), common BMP fatty acyl chains^75^ (Fig. 4J). Meanwhile, palmitate (16:0) was the primary PG/BMP fatty acyl group (**Fig. S10E**), while it comprised a much smaller portion of total BMP signal (Fig. 4J). BMP content in lipofuscin reflects the involvement of late endosomes/lysosomes in lipofuscin formation. BMP is predominantly enriched in these structures, where it regulates formation of intraluminal vesicles (ILVs)^75,76^.

### PPT1 activity is predictive of lipofuscin formation

PPT1 protein exhibited significant increases with age in WT and was expectedly absent in PPT1 KO brains (Fig. 5A and Fig. 5B). However, this increase in PPT1 protein was not accompanied by an increase in activity. At the level of the total brain, PPT1 activity exhibited a modest decline with age (Fig. 5C), while examinations of specific enzymatic activity (accounting for PPT1 protein levels) revealed a significant progressive decline with age (Fig. 5D). These data suggest that PPT1 enzyme deficiency may also contribute to lipofuscin formation in WT brain.

To evaluate if PPT1 specific activity is a determinant of lipofuscin load independent of age, we quantified lipofuscin in WT and *Ppt1* +/− (heterozygotes) at 4 months of age with the QUINT pipeline. We hypothesized *Ppt1* +/− brains would exhibit partial PPT1 activity and thus intermediate lipofuscin load between the WT and KO models. We found that in this holdout validation of our line of best fit (Fig. 1C) with 4-month-old WT, age was an accurate predictor of brain lipofuscin load, underestimating measured load values by just 1.1% (Fig. 5E). Surprisingly, we found that 4-month-old *Ppt1* +/− mice had very similar loads to 4-month-old WT (Fig. 5E). We thus examined PPT1 protein levels and enzyme activity in 4-month-old animals of each genotype. Although *Ppt1* +/− mice had 58% PPT1 protein compared to WT (Fig. 5F and Fig. 5G), total PPT1 enzyme activity was not significantly different (Fig. 5H). The resulting increase in PPT1 specific activity (Fig. 5I), implicates genetic compensation in response to *Ppt1* heterozygosity. Together, these data indicate that PPT1 enzyme activity level, distinct from PPT1 protein level, is a strong negative predictor of lipofuscin formation, independent of age-related factors.

## Discussion

Despite the ubiquitous presence of lipofuscin across many tissues and species, the spatiotemporal accrual and detailed composition of this enigmatic autofluorescent storage material has remained unclear. Here, we detail the linear neuroanatomical deposition of lipofuscin with aging and NCL progression in mouse brain. These data reveal regional vulnerability of cortical, thalamic, and cerebellar areas. This first-of-its-kind brain atlas of lipofuscin (https://lipofuscinatlas.yale.edu) can guide careful interpretation of fluorescence studies of brain tissues and interrogation of the aging or *CLN1* brain.

We elucidated the organellar origin and molecular composition of purified lipofuscin with ultrastructural analysis and multimodal mass spectrometry. Lipofuscin predominantly consists of proteins of lysosomal, mitochondrial, and synaptic origin, and is remarkably similar in protein composition in aging and NCL. This unique composition is corroborated by the presence of organelle-specific lipids, BMP and cardiolipin. Ultrastructure of purified lipofuscin granules reflects these contents; autofluorescent structures are revealed to be autophagy-lysosome components and damaged mitochondria along with canonical electron-dense aggregates. These findings can explain why lipofuscin was never identified as a *de novo* substance of uniform composition, since autofluorescence occurs on multiple dysfunctional organelles. We also demonstrate that PPT1 deficiency is sufficient to cause mitochondrial deficits in the absence of lipofuscin. These data support the hypothesis that lipofuscin forms due to deficient autophagy of oxidatively damaging and dysfunctional mitochondria, which in turn leads to the accumulation of secondary lysosomes and their limit digests^27,28,30^. It is noteworthy that other lysosomal storage diseases do not accrue lipofuscin, which strongly indicates that lysosomal deficits alone are not sufficient to produce this phenotype. Together, our findings support the mitochondrial-lysosomal axis theory of aging^28^, which posits that interrelated mitochondrial and lysosomal damage irreversibly leads to functional deficits and eventual death of postmitotic cells.

Molecular identification of total brain and lipofuscin lipid content is congruent with other studies^77^ and reveals many BMP metabolites to be lipofuscin-specific biomarkers. BMP stimulates lipid-degrading enzymes on ILVs in concert with saposins A-D (SAP) and maintains a lysosomal environment that supports the function of protein hydrolases^75^. BMP (especially BMP 22:6/22:6) is implicated in several lysosomal storage disorders and neurodegenerative diseases^74,75^. BMP was also recently identified as an aging biomarker in humans^78^, a finding which may be explained, in part, by the accumulation of lipofuscin-positive cells with age. Recent identification of the *CLN5* causal gene product as a possible BMP synthase^79,80^ also highlights the interplay of NCL-related proteins and lipid homeostasis as a critical axis of lysosomal function. Changes in TPP1 (*CLN2*) and CATD (*CLN10*) enzyme activity with time and in *CLN1* suggest coregulation of NCL proteins and a critical contribution of this lysosomal storage disorder network to natural aging processes.

Polyunsaturated fatty acids (PUFAs) and unsaturated OxPE metabolites are enriched in lipofuscin. Additionally, lipid droplets in aged WT lipofuscin (and congruent enrichment of triglycerides) suggests alteration in beta-oxidation of lipids by mitochondria. These data support the contribution of membrane lipid oxidative damage^27^ and PUFA peroxidation to lipofuscin formation^81^. Oxidation of PUFAs can induce the formation of Schiff base adducts and cyclic aromatic structures. We suggest that polyunsaturated OxPE derivatives may therefore be sources of lipofuscin autofluorescence akin to retinal A2E^82^. Further work is needed to understand the effects of lipid accumulation in lipofuscin on global lipid metabolism and energy homeostasis.

Strikingly, almost all age- and *CLN1*- lipofuscin proteins were classified as S-acylated. These include many PPT1 substrates, which exhibit elevated S-acylation in PPT1 KO brain^7^. PPT1 substrates have long been hypothesized to be protein constituents of *CLN1* lipofuscin^52^. We now provide the first proof of this seminal hypothesis. Our findings that lipofuscin continuously accrues with time and that PPT1 specific activity is a determinant of lipofuscin load suggest that cellular mechanisms to abrogate persistent S-acylation are insufficient across the brain. Indeed, we observe that lipofuscin proteins are intact although lysosomal enzymes are active in lipofuscin preparations. We speculate that removal of S-acyl modifications enables the degradation of protein substrates, which otherwise become sequestered in lipofuscin structures. This raises important questions regarding how protein, fatty acid, and acyl-Coenzyme A turnover are regulated by S-acylation and how aberrant lipidation contributes to age-related neuronal and metabolic dysfunction. Overall, our results indicate that S-acylation is a hitherto unappreciated fundamental contributor to aging.

## Methods

### Experimental model details

WT (C57BL6/J) and PPT1 KO (B6;129-*Ppt1*^*tm1Hof*^/J) mice^9^ were purchased from The Jackson Labs. PPT1 KO mice were successively backcrossed to C57BL6/J to obtain a congenic C57BL6/J background. Independent single nucleotide polymorphism (SNP) genome scanning analysis was performed by The Jackson Labs to verify homogenous C57BL6/J genetic background. Mice heterozygous for *Ppt1* (*Ppt1* +/−) were obtained by crossing WT (*Ppt1* +/+) and PPT1 KO (*Ppt1* −/−) animals. Animal care and housing in compliance with the Guide for Care and Use of Laboratory Animals^90^ were provided by the Yale Animal Resource Center (YARC). Mice were maintained on a 12-hour light/dark cycle with *ad libitum* access to water and standard chow. PPT1 KO mice were aged to 2, 4, and 7 months (referred to as PPT1–2, PPT1–4, PPT1–7, respectively) and WT mice were aged to 2, 12, 18, and 24 months (referred to as WT-2, WT-12, WT-18, WT-24, respectively) to capture multiple stages of lipofuscin accumulation and disease and aging progression. Experimental animals were sex-matched and sex-balanced wherever possible. All experimental protocols involving animals were approved by the Institutional Animal Care & Use Committee (IACUC) at Yale University.

Human brain sections from dorsolateral prefrontal cortex (dlPFC) were obtained from Dr. Pallavi Gopal and the Yale Pathology Department, Center for Human Brain Discovery Brain Bank. HEK293T cells were obtained from American Type Culture Collection.

### Tissue preparation for histology

Animals were deeply anaesthetized with 100% isoflurane and transcardially perfused with ice-cold 0.9% saline (w/v) with 0.05 mg/mL heparin sodium salt (H3393, Sigma-Aldrich), followed by filtered 4% (w/v) paraformaldehyde (PFA) (158127, Sigma-Aldrich) in PBS buffered to pH 7.4 with NaOH. Brains were postfixed and cryoprotected at 4°C in 4% (w/v) PFA in PBS for 24–48 hours, then 15% (w/v) sucrose in PBS for 24 hours, then 30% (w/v) sucrose in PBS for 24 hours. Brains were embedded in Tissue-Tek OCT medium (4583, Sakura) and stored at −80°C until sectioning. Brains were sectioned (30 *μ*m) with a cryostat (Leica CM1850). Microscope slides were coated with 2% gelatin from porcine skin (G1890, Sigma-Aldrich) with 0.1% chromium (III) potassium sulfate dodecahydrate (243361, Sigma-Aldrich). Four alternate sagittal sections (~240 *μ*m apart) were thaw-mounted onto each microscope slide. Sections were stored at −20°C prior to use.

### Microscopy of in situ lipofuscin autofluorescence

Alternate sagittal sections (approximately represented by Allen Mouse Brain Atlas sagittal planes (13–19 of 21) were selected for QUINT histological analysis (2 male and 2 female mice/genotype/timepoint; 4 sections/mouse). Coverslips were mounted with antifade mounting medium with DAPI (H-1200, Vectashield) and sealed with clear nail polish. Whole brain fluorescent images were acquired with a VS200 slide scanner (Olympus) in the DAPI and FITC channels at 20X in an appropriate DAPI z-plane determined by instrument autofocus settings. Hippocampal formation and cortex z-stack images were acquired on the slide scanner at 40X. Hippocampus subregion images were acquired with a laser scanning confocal microscope (LSM800, Zeiss) with a 63X oil-immersion objective and DAPI and AF488 lasers.

### QUINT

To generate a spatiotemporal atlas of lipofuscin distribution in PPT1 KO and WT mouse brains across disease progression and aging, we applied a modified QUINT histology pipeline^91,92^, which combines the following software programs. The investigator was masked to genotype and age for all images prior to analysis.

1. *Cell detection and lipofuscin signal segmentation in QuPath*. Native full resolution 20X whole brain VSI images were loaded onto QuPath^84^ software version 0.02.3. A cell detection algorithm was run using DAPI signal with a constant threshold, min/max area, and sigma. Cell detection false positives that fell outside of the intact tissue slice were manually removed. An artificial neural network object classifier (ANN_MLP) was trained to segment lipofuscin positive cells. The training image set contained randomly selected example region images from each timepoint and genotype (Fig. 1A) to ensure accurate signal-to-noise discrimination across the full range of possible lipofuscin signal intensity and distribution. Additional regions from the training image set were added until the performance of the network ceased to improve, then the object classifier was applied to segment lipofuscin signal in all experimental images. Images were exported prior to cell detection and after cell detection for inputs at step 2 and 4, respectively.
2. *Allen Mouse Brain Atlas registration in QuickNII*. PNG Images exported from QuPath prior to cell detection were downsampled by a factor of 12 and grouped into an XML file using FileBuilder. The XML was imported into QuickNII-ABAMouse-v3 2017 software for spatial registration of brain slice images onto the Allen Mouse Brain Atlas CCFv3^87^. Using QuickNII, the anatomical landmarks of each experimental section were used to identify the most accurate corresponding reference atlas section. Each image was then superimposed with and anchored to this reference atlas in three-dimensional space, as described by Puchades et al., 2019^85^. The reference atlas overlay was then linearly transformed to fit the proportions and spatial positioning of the experimental section. Following reference space assignment and linear transformation for all experimental sections, reference atlas overlays were saved as a JSON file for input at step 3. Note that Allen Mouse Brain Atlas registration at this step has several specific limitations to the inclusion of fine neuroanatomical regions. For example, in the hippocampus the *stratum pyramidale*, *stratum oriens*, *stratum radiatum*, and *stratum lacunosum-moleculare* areas are grouped into a single measured region for fields CA1, CA2, and CA3. Similarly, in the cerebellum, the granular layer and the molecular layer are averaged for each vermal region. The layers of the main olfactory bulb and the accessory factory bulb are also grouped together into single regions by this software.
3. *Non-linear alignment of reference atlases for analysis of fine neuroanatomical regions in VisuAlign.* Sagittal brain atlas overlays for all experimental sections were imported into VisuAlign software v0_9 (VisuAlign, RRID:SCR_017978), which allows the anatomical region outlines to be subject to manipulation. Non-linear transformation of the atlas overlay outline was performed to closely align the reference atlas to the true anatomy of the experimental section. Images were saved as FLAT and PNG files and as a master record with alignment transformations in a JSON file for data compilation in the next step.
4. *Quantification of region areas and lipofuscin signal with Nutil.* The “Quantifier” function of Nutil^86^ was used to aggregate segmented lipofuscin signal data from step 1 with transformed anatomical atlas overlays in step 3 to obtain a spatial quantification of lipofuscin load (defined as object pixels/region pixels). Nutil outputs include region pixels, object pixels, and load for 1325 fine anatomical regions (including parent regions) and 10 gross custom regions (CTX: cortex; OLF: olfactory; HPF: hippocampal formation; STR/PAL: striatum/pallidum; TH: thalamus; HY: hypothalamus; MB/P/MY: midbrain/hindbrain/medulla; CB: cerebellum; FT: fiber tracts; VS: ventricular system).
5. *Collation, quantification, and visualization of lipofuscin load data.* The individual Nutil export files for each brain section were collated in Excel (Microsoft). The average lipofuscin load was determined for each series of 4 brain sections per biological replicate, excluding zero values which indicate that the region was not present in that section. Graphed heat map values were generated in GraphPad Prism software version 10.1.1. Heatmaps represent the average load of each biological replicate, excluding replicates where a load value was not obtained for that region. Total brain lipofuscin load (Fig. 1C) is the sum of loads across all custom regions with standard deviation error bars.

Coronal heatmaps were generated according to procedures on the Mouse-Brain-Heatmap-Website GitHub repository (https://github.com/MXHend/Mouse-Brain-Heatmap-Website?tab=readme-ov-file). Raw Nutil outputs were imported into the Shiny application NutilToUsable (https://github.com/DaniellaDeWeerd/NutilToUsable). “Mouse” was selected as the x-axis variable, “daughter” as the y-axis variable, and “parent/special” as the segmenting x-axis variable. No regions were removed. The lipofuscin load color scale range was set to 0–0.2 for maximum contrast across all conditions, although several PPT1 KO values fell above 0.2 (shown in maximum scale color). Generated mouse brain heatmap table and color values were input into the Mouse_Brain_Heatmap Shiny applications website (https://github.com/vari-bbc/Mouse_Brain_Heatmap). The plotted Allen Mouse Brain Atlas coronal section was selected as “Figure Number” 71 of 132. Data were obtained for the right hemisphere and reflected across the midline for purposes of visualization. Imaging was performed in the sagittal plane, thus values for temporal regions were not obtained (represented in grey). Coronal heat map color values represent the average of *n =* 4 sex-matched biological replicates/genotype/timepoint.

Lipofuscin-positive cell intensity histograms were generated by binning all lipofuscin-positive cell detections by raw intensity (33 bins of 2000 A.U. width; *n =* 4 sections collated for each of *n =* 4 biological replicates).

Lipofuscin atlas load data and coronal heatmaps for each genotype and timepoint are available as a web tool at https://lipofuscinatlas.yale.edu/.

### Immunohistochemistry

Mounted sagittal brain sections were equilibrated in 1X PBS (pH 7.4; 5 min), washed in 1 X PBS with 0.25% Triton-X (PBS-X; 2 x for 5 min) then blocked in 5% normal goat serum (NGS) in PBS-X (RT, 1 hr). Sections were incubated in 1° antibody in 5% NGS in PBS-X overnight at 4°C in a humidified chamber to prevent solution evaporation. The next day, slides were washed in PBS-X (3x for 5 min), incubated in 2° antibody (1.5 hrs, RT, protected from light), washed in PBS-X (3x for 5 min), then dried while protected from light. Coverslips were mounted with antifade mounting medium with DAPI (H-1200, Vectashield) and sealed with clear nail polish. Antibody conditions are outlined in Table 1.

### Primary culture and immunocytochemistry

Primary cortical neurons from WT and PPT1 KO mice (P0) were cultured on coverslips^93^. At DIV 14, neurons were fixed with 4% (w/v) buffered PFA with 4% (w/v) sucrose, washed in 1X PBS and blocked in 3% (v/v) goat serum in 1X PBS at room temperature. Neurons were incubated in 1° antibodies overnight at 4°C, then in Alexa-conjugated 2° antibodies at 4°C. Coverslips were mounted onto slides with antifade mounting medium with DAPI (H-1200 Vectashield) and sealed with clear nail polish. Confocal fluorescent images were obtained with a laser scanning confocal microscope (LSM800, Zeiss) with a 63X oil-immersion objective. Antibody conditions are outlined in Table 1.

### Immunohistochemistry & immunocytochemistry image analysis

Immunohistochemistry (IHC) and immunocytochemistry (ICC) images were analyzed with Fiji ImageJ^88^ version 214.0/1.54f.

ICC images were converted to 8-bit and average intensity projections were generated from z-stacks. 25 × 25 *μ*M regions of interest (ROIs) were centered on neuronal nuclei in the DAPI channel of de-identified images to capture the cell body. To quantify protein expression, mean gray value was measured on a scale of 0–255. For each genotype, plotted values are the average of *n =* 3 independent primary neuronal cultures with *n =* 5 images/culture.

IHC images were converted to 8-bit and average intensity projections were generated from z-stacks, then subjected to background correction using ImageJ command ‘Subtract Background’ with a rolling-ball radius of 100 px to correct for uneven illumination due to holes in the tissue. The entire hippocampal formation was isolated as an ROI for quantification, while CA1 is shown in representative images. To quantify protein expression, mean gray value was measured on a scale of 0–255. Plotted values are the average of *n =* 4 biological replicates/genotype with *n =* 4 sections/replicate.

### Lipofuscin purification from murine brain

Mice were deeply anaesthetized with 100% isoflurane (Henry Schein Animal Health) using an open drop method^94^ and sacrificed by cervical spine dislocation followed swiftly by decapitation. Brains were excised as previously described^95^. Individual fresh brains were added to 5 mL ice cold 320 mM sucrose (Sigma-Aldrich) in Buffer A (10 mM N-[2-Hydroxyethyl] piperazine-N’-[2-ethanesulfonic acid] (HEPES; American Bio), pH 7.4, 1 mM phenylmethylsulfonyl fluoride (PMSF; American Bio), 1 *μ*g/mL aprotinin (Sigma-Aldrich), 1 *μ*g/mL leupeptin (American Bio), 1 *μ*g/mL pepstatin A (Thermo Scientific), cOmplete, EDTA-free Protease Inhibitor Cocktail Tablets (Roche)) in a glass Dounce homogenizer on ice. Brains were homogenized with 12 up-down passes at 500 rpm with a Teflon-coated pestle (Thomas Scientific). Small aliquots of brain homogenates were frozen at −80°C for immunoblotting and enzyme activity analyses. Lipofuscin was purified from fresh brain homogenates using methods adapted from Boulton and Marshall (1985), Schutt et al. (2002), and Ottis et al. (2012)^18,20,22^. Briefly, whole brain homogenates were combined from 1 male and 1 female mouse for each genotype and age and subjected to low-speed centrifugation (100 × g, 7 min, 4°C). The resultant supernatant was centrifuged to pellet lipofuscin granules (6000 × g, 10 min, 4°C). The pellet was resuspended in 1 mL 320 mM sucrose in Buffer A and the suspension was overlaid onto a discontinuous gradient consisting of variable concentrations of sucrose in Buffer A: 1.0 M, 1.2 M, 1.4 M, 1.50 M, 1.55 M, 1.6 M, 1.8 M (1.5 mL each; except for 1.2 and 1.4 – 2 mL). To achieve sample fractionation, gradients were ultracentrifuged in a swinging-bucket rotor (100,000 × g, 1 hr, 4°C; SW40 Ti, Beckman Coulter). Fractions at each sucrose interface were sequentially collected and subjected to fluorescence spectroscopy. The crude lipofuscin fraction was diluted with 0.5 mL 1X PBS and pelleted (6000 × g, 10 min, 4°C). The pellet was resuspended in 1 mL 320 mM sucrose in Buffer A and overlaid on a simplified discontinuous sucrose gradient: 1.0 M, 1.2 M, 1.4 M, 1.50 M. To achieve greater separation around the interfaces of interest, the second gradient consisted of larger volumes (3 mL each, except for 1.50 M – 1 mL) and was subjected to ultracentrifugation as above. Fractions at each sucrose interface were collected and the pure principal lipofuscin fraction was identified with fluorescence spectroscopy.

Crude and pure sucrose gradient interface fractions were loaded into a black clear-bottom 96-well plate (75 *μ*L; technical triplicates; 16305, Thermo Scientific). Autofluorescent spectra were collected at an excitation wavelength of 370 nm to achieve the characteristic emission maximum^18,20^. Emission spectra were measured from 400–700 nm on a BioTek Gen5 Microplate Reader (Agilent). The fraction with the highest fluorescence emission peak was considered the principal crude or pure lipofuscin fraction. Visually, lipofuscin fractions were a yellow-brown material and were typically present at the 1.0–1.2 M sucrose interface following ultracentrifugation.

### Lipofuscin bright field microscopy & morphology quantification

Pure lipofuscin fractions were spread on microscope slides coated with 2% gelatin from porcine skin (G1890, Sigma-Aldrich) with 0.1% chromium (III) potassium sulfate dodecahydrate (Sigma-Aldrich, 243361) and air-dried. Slides were incubated in freshly prepared and filtered 1% (w/v) solvent black 3 (SB3; Sigma-Aldrich, 199664) in 70% ethanol for 5 minutes. Slides were destained with a gentle dH_2_O rinse, then air-dried prior to mounting a coverslip with antifade mounting medium (Vectashield, H-1000).

Bright-field SB3-stained granule images were obtained on a BioTek Lionheart FX Automated Microscope (Agilent) at 60X (*n =* 5 collated visual field images per sample; *n =* 4 sex-matched biological replicates per genotype and condition). Using Fiji ImageJ^88^ version 214.0/1.54f, images were converted to 8-bit and thresholded from 0–160 using the *Otsu* algorithm. The *analyze particles* function (exclude edges; include holes) was used to capture lipofuscin granule Feret’s diameter and circularity. A threshold of 0.04–5.00 *μ*M^2^ was set based on pilot observations to avoid capturing background and aggregated particles. Statistical comparisons were made with a nested one-way ANOVA with Tukey’s correction for multiple comparisons.

### Lipofuscin autofluorescence microscopy

To image lipofuscin granule autofluorescence, pure lipofuscin fractions were dropped onto a gelatin-coated microscope slide and immediately mounted with an equal volume of mounting medium (H-1000, Vectashield) and a glass coverslip. Lipofuscin granules were then imaged with a BioTek Lionheart FX Automated Microscope (Agilent) at 60X in the FITC channel.

### Electron microscopy

#### Negative staining of purified lipofuscin:

Purified lipofuscin granules were negatively stained with 2% uranyl acetate on glow discharged 300-mesh carbon-coated Copper grids (EMS) and imaged on a 200 kV Tecnai F20 Transmission electron microscope (ThermoFisher Scientific). Images were acquired using a side-entry AMT NanoSprint12 12-megapixel camera (AMT Imaging) at 15,300–29,700x.

#### Positive staining of purified lipofuscin:

Lipofuscin granules stored at −20°C were thawed on ice and fixed with 2.5% glutaraldehyde (Electron Microscopy Sciences [EMS], Hatfield, PA, USA) buffered in 0.1 M sodium cacodylate, pH 7.4. The granules were pelleted, and the supernatant was carefully removed. The pellet was resuspended in 2% molten agar buffered in 0.1 M sodium cacodylate buffer. The lipofuscin-agar suspension was allowed to solidify on ice and transferred to glass vials for the following solution changes. The samples were rinsed with 0.1 M sodium cacodylate and then post-fixed with 1% osmium tetroxide (EMS) for one hour. The lipofuscin-agar pellets were then rinsed in 0.1 M sodium cacodylate followed by HPLC water. To facilitate epoxy resin infiltration, the samples were treated with an increasing ethanol gradient followed by a transition to propylene oxide (EMS). The samples were infiltrated overnight in a 1:1 (v/v) solution of propylene oxide:EPON 812 epoxy resin (EMS). The following day, the pellets underwent two changes of pure EPON 812 epoxy resin. The pellets were embedded in fresh EPON 812 epoxy resin which was polymerized at 60°C for 24 hrs until solid.

Ultrathin (60 nm) sections were cut on an Ultracut UC7 Ultramicrotome (Leica) and mounted on formvar-coated Nickel mesh grids. The grids were post-stained in 2% uranyl acetate and Reynold’s lead citrate.

#### Brain tissue preparation for electron microscopy of in situ lipofuscin:

PPT1 KO (7-months) and WT (24-months) mice were deeply anaesthetized with 100% isoflurane and transcardially perfused with pre-warmed 1X PBS (37°C) followed by filtered 4% (w/v) paraformaldehyde (PFA) (158127, Sigma-Aldrich) in PBS buffered to pH 7.4 with NaOH. The brain was excised and immersed in 2.5% glutaraldehyde, 2% paraformaldehyde, buffered by 0.1 M sodium cacodylate (pH 7.4) on a shaker (30 min, RT). The tissue was sectioned sagittally using a scalpel. Small regions (~1×1×2 mm^3^) containing field CA3 of hippocampus and somatomotor or somatosensory cortex were isolated and incubated in fixative for an additional hour (RT), then overnight (4°C).

Samples were rinsed (3X, 10 min each) in 0.1 M sodium cacodylate buffer, then post-fixed in 1% osmium tetroxide, 0.8% potassium ferrocyanide, 0.1 M sodium cacodylate. After rinsing with 0.1 M sodium cacodylate and HPLC water, the samples underwent en bloc staining in 2% uranyl acetate and were rinsed with HPLC water. Samples were dehydrated in sequential solutions of 50%, 70%, 90%, and 100% anhydrous ethanol and transitioned into a 1:1 (v/V) solution of 100% ethanol:propylene oxide. After two changes of 100% propylene oxide, the tissues were transitioned into a mixture of 1:1 (v/v) propylene oxide:EPON epoxy resin which was allowed to infiltrate overnight. The following day, the tissues underwent two changes in fresh EPON epoxy resin and were embedded in EPON. The resin blocks were polymerized overnight at 60°C.

Ultrathin (60 nm) sections were cut on a UC7 Ultramicrotome (Leica) and mounted on formvar-coated Nickel mesh grids (EMS). The grids were post-stained with 2% uranyl acetate and Reynold’s lead citrate.

#### Electron microscopy imaging of purified lipofuscin and brain tissue:

Sample grids were imaged on a Tecnai 12 BioTwin Transmission electron microscope (ThermoFisher Scientific, Hillsboro, Oregon) operating at 80 kV. Images were acquired on a NanoSprint15 MKII camera (AMT Imaging Systems, Woburn, MA).

All reagents (except ethanol and HPLC water) were obtained from Electron Microscopy Sciences (EMS, Hatfield, PA).

### Flow cytometry

Flow cytometry of dissociated single lipofuscin particle suspensions was conducted on a 7 laser Bigfoot cell sorter from Thermo Fisher Scientific. Small particles were identified using the forward scatter (FSC) small particle detector on the 405 nm laser vs. side scatter (SSC) as well as thresholding on the 405nm-455/14 parameter. Size beads from Thermofisher (F13839) were run to establish a reference gate for lipofuscin particles approximately 1.0 *μ*m in diameter. Spectral energy plots revealed autofluorescence in the 455/14 channel and the 473/15 channel of the 405 nm laser in lipofuscin samples compared to the quenched sample and buffer controls. Autofluorescent gates were set within the 455/14 channel in reference to a quenched sample (see below). A minimum of 50,000 events were captured per sample above the threshold. Data were analyzed with FCS Express 7 Research Edition (De Novo Software).

To quench autofluorescence, purified lipofuscin was incubated with or without a 2X volume of Solvent Black 3 (Sigma-Aldrich; Cat # 199664 in 70% ethanol; passed through 0.2 *μ*m filter) for 5 min, 21°C, diluted in sort buffer (1 X phosphate buffered saline, 1 mM phenylmethylsulfonyl fluoride (PMSF; American Bio), 1 *μ*g/mL aprotinin (Sigma-Aldrich), 1 *μ*g/mL leupeptin (American Bio), 1 *μ*g/mL pepstatin A (Thermo Scientific), cOmplete, EDTA-free Protease Inhibitor Cocktail Tablets (Roche)), pelleted at 6,000 x g, 5 min, 4°C, then resuspended in sort buffer.

### Quantitative immunoblotting

SDS-PAGE and western blots were performed using standard procedures and the antibody conditions outlined below. Western blot images were collected on an Odyssey XF Imager (LI-COR), and densitometry values were obtained using Image Studio software version 5.2.5 (LICOR). Antibody conditions are outlined in Table 1.

### CRISPR-Cas9-mediated genetic deletion in HEK293T cells

CRISPR-Cas9 was used to generate *PPT1* knockout HEK293T cells. A guide RNA (gRNA) oligonucleotide designed to target *PPT1* [CACCGCGCCGCTGCCGTTGGTGATC] was cloned into a pSpCas9(BB)-2A-GFP (PX458) (Addgene, 48138) backbone according to Ran et al., 2013^83^. Successful insertion of the gRNA was verified by sequencing of the plasmid with a U6 promoter primer [GACTATCATATGCTTACCGT]. HEK293T cells were cultured in 6-well plates in Dulbecco’s Modified Eagle Medium (DMEM; Gibco, 11965–092) with 10% fetal bovine serum (FBS; Gibco, A56708–01) and 1% penicillin/streptomycin (Gibco, 15140–122) at 37°C, 5% CO_2_. When cells reached 70% confluence, they were transfected with 2 *μ*g of plasmid DNA with GenePORTER 3000 transfection reagents (amsbio, AMS.T203007) according to the manufacturer’s protocol.

After 48 hours of incubation, successfully transfected GFP-positive cells were sorted by flow cytometry into 24-well plates (4,000 cells/well). Following clonal expansion, colonies were manually picked and passaged as discrete clonal cultures. To confirm targeted disruption of *PPT1*, genomic DNA was extracted from clonal HEK293T pellets (KAPA Express; Roche, KK7102) and PCR amplification of the region was performed using primers (Forward (F): [TTTTGATTCACCGCAGAGGG], Reverse 1 (R1): [ATCCCATGCCAGATCACCAA], Reverse 2 (R2): [GTAAAACTTCAACGCCGTGC]) that flanked (F + R2) or included (F + R1) the CRISPR site. PCR products (F + R2) were purified (QIAquick PCR Purification Kit; Qiagen, 28104) and sequenced to confirm the presence of indels. *PPT1* disruption was also functionally validated by assaying PPT1 enzyme activity in cell pellets (see “PPT1 Enzyme Activity” below) as shown in **Fig. S5A**.

### Mitochondrial function assay

To measure metabolic flux, HEK293T control and PPT1 KO cell lines were cultured in each half of a Seahorse 96-well plate (Agilent, 103794–100) at a seeding density of 2 × 10^4^ cells/well. HEK293T cells were grown in 100 *μ*L Dulbecco’s Modified Eagle Medium (DMEM; Gibco, 11965–092) with 10% fetal bovine serum (FBS; Gibco, A56708–01) and 1% penicillin/streptomycin (Gibco, 15140–122) for 24 hours. The day prior to the assay, medium in 96-well plates was replaced with 100 *μ*L substrate-limited growth medium (Seahorse XF DMEM (Agilent, 103575–100), 0.5 mM glucose, 1.0 mM glutamine, 1% FBS, 0.5 mM L-carnitine, pH 7.4), and incubated overnight. On the day of the assay, growth medium was replaced with 180 *μ*L substrate-limited assay buffer (Seahorse XF DMEM, 2 mM glucose, 0.5 mM carnitine, pH 7.4), and incubated for 1 hour in a CO_2_-free incubator at 37°C. Seahorse Cell Mito Stress Test injection reagents (Agilent, 103015–100) were reconstituted with assay buffer at pH 7.4. Immediately prior to loading the plate to read on a Seahorse XFe96 instrument (Agilent), medium in each well was replaced with 150 *μ*L substrate-limited assay medium + 30 *μ*L 0.17 mM bovine serum albumin (BSA; Agilent, 102720–100). Using an assay cartridge (Agilent, 103793–100), ten measurements of oxygen consumption rate (OCR) and extracellular acidification rate (ECAR) were taken to establish a baseline, followed by three measurements after each of three sequential injections of mitochondrial inhibitors: oligomycin A (1.5 *μ*M), carbonyl cyanide-4 (trifluoromethoxy) phenylhydrazone (FCCP) (1.0 *μ*M), and rotenone/antimycin A (0.5 *μ*M/0.5 *μ*M). Oligomycin A is an ATP synthase (complex V) inhibitor. FCCP is an uncoupling agent that collapses the proton gradient and disrupts mitochondrial membrane potential. Rotenone and antimycin A are complex I and complex III inhibitors, respectively, which together abolish mitochondrial respiration. Hoescht 33342 solution (Thermo Fisher, 62249), a nuclear counterstain, was spiked in with the rotenone/antimycin A injection and used to determine cells/well at the completion of the assay. OCR and ECAR readings were then normalized to the number of cells/well.

Basal respiration was calculated as the difference between baseline OCR prior to oligomycin injection and after rotenone/antimycin A injection (non-mitochondrial respiration rate). Maximal respiration was the difference between the maximum rate measurement after FCCP injection and non-mitochondrial respiration rate. Proton leak was calculated as the difference between the minimum rate measurement after oligomycin injection and non-mitochondrial respiration rate. ATP production was the difference in OCR before and after oligomycin injection. Spare respiratory capacity percentage was calculated as the ratio of maximal respiration to basal respiration. Coupling efficiency was the ratio of ATP production to basal respiration. Wells with fewer than 5,000 cells were excluded from analyses due to insufficient cell growth. Representative data (**Fig. S5**) was replicated in two independent experiments.

### Label-Free Quantification Mass Spectrometry (LFQ-MS)

Pure lipofuscin fractions were diluted with PBS and pelleted, then washed in ultrapure distilled H_2_O (RPI) to remove sucrose and protease inhibitors (6000 x g, 10 min, 4°C). The washed lipofuscin pellet was frozen at −80°C prior to mass spectrometry. Label-Free Quantification Mass Spectrometry (LFQ-MS) was performed at the Yale Mass Spectrometry & Proteomics Resource of the W.M. Keck Foundation Biotechnology Resource Laboratory with the support of the Yale/NIDA Neuroproteomics Center.

Lipofuscin pellets were dissolved in 0.1% RapiGest SF (Waters Corporation, SKU: 186001861) in 50mM ammonium bicarbonate, reduced with DTT (final concentration of 4.5 mM, 37°C, 30 min.) and alkylated with iodoacetamide (final concentration of 10 mM, room temperature, 30 min., in the dark). Reduced and alkylated proteins were then treated with PNGase (37°C, overnight, Promega, V4831) followed by digestion with Lys-C (1:50 enzyme:protein ratio; 37°C, 6.5 hours) and subsequently with trypsin (1:50 enzyme:protein ratio; 37°C, overnight). The solution was then acidified with 20% trifluoroacetic acid (TFA; pH <2, 37°C, 45 min.; Thermo Fisher Scientific, 28901) and desalted with a BioPureSPN PROTO 300 C18 Mini desalting column (The Nest Group, HUMS18V). The effluents from the de-salting step were dried and re-dissolved in 5 *μ*l 70% formic acid (FA) and 35 *μ*l 0.1% TFA. Protein concentration was measured via Nanodrop and samples were diluted to 0.05 *μ*g/*μ*l with 0.1% TFA in a 98:2% water:acetonitrile buffer. To check for retention time variability and normalization during LFQ data analysis, a 1:10 dilution of 10X Pierce Retention Time Calibration Mixture (Thermo Fisher Scientific, 88321) was added to each sample prior to injecting on the mass spectrometer.

LFQ-MS was performed on a Thermo Scientific Q-Exactive plus Mass spectrometer connected to a Waters nanoACQUITY UPLC system equipped with a Waters Symmetry^®^ C18 180 μm × 20 mm trap column and a 1.7-μm, 75 μm × 250 mm nanoACQUITY UPLC column (35°C). 5 *μ*l of each 0.05 *μ*g/*μ*l digest were injected in block randomized order (each biological replicate injected in technical triplicate). To ensure a high level of identification and quantitation integrity, a resolution of 70,000 was utilized for MS and 20 MS/MS spectra were acquired per MS scan using higher-energy collisional dissociation (HCD). All MS (Profile) and MS/MS (centroid) peaks were detected in the Orbitrap. Trapping was carried out for 3 min at 5 *μ*l/min in 99.5% Buffer A (0.1% FA in water) and 0.5% Buffer B (0.075% FA in acetonitrile (ACN)) prior to eluting with linear gradients that reached 25% B at 140 min, 40% B at 165 min, and 90% B at 170 min. Four blanks (2× 100% ACN wash, then 2x Buffer A wash) were injected after each sample to ensure against sample carryover.

As previously^7,96^, chromatographic/spectral alignment, mass spectral peak picking and filtering (ion signal > 3X standard deviation of noise), and quantification of proteins and peptides were performed with Progenesis QI software (Nonlinear Dynamics, version 4.2). A normalization factor was calculated to account for sample load and ionization differences across technical triplicate injections. An in-house Mascot search engine (2.7) was used to carry out protein identification against the Swiss Protein database with taxonomy restricted to *Mus musculus*. Two missed tryptic cleavages were allowed, precursor mass tolerance was set to 10 ppm, and fragment mass tolerance was set to 0.02 Da. The protein identification significance threshold was set based on a False Discovery Rate (FDR) of 2%. Protein abundances were calculated from the sum of all non-conflicting peptide ion ID assignments for a specific protein on each run, then normalized to Pierce Retention Time Calibration Mixture and spectral counts. Proteins with 2+ unique peptides and a confidence score > 39 were included in the lipofuscin proteome for further analysis. Proteins are listed in UniProt Protein ID nomenclature.

Protein abundance comparisons were considered significant with a fold-change of 1.5 and a p-value < 0.05 by two-tailed unpaired t-test. Ingenuity Pathway Analysis (IPA; Qiagen) was conducted to determine represented canonical pathways. Organelle enrichment was determined by annotating proteomic data by primary localization in the Human Protein Atlas^55^ and quantifying the annotated proteins per category/total detected proteins in lipofuscin versus the expected whole brain ratio in the Human Protein Atlas. Mitochondrial compartment annotation (MOM: mitochondrial outer membrane; IMS: intermembrane space; MIM: mitochondrial inner membrane) was performed using MitoCarta 3.0^56^ as a reference. The ratios of proteins from each mitochondrial compartment/total proteins were compared by two-tailed unpaired t-test for lipofuscin vs. mitochondrial isolates from different brain regions (spinalcord, brainstem, cerebellum, cerebrum) detailed in MitoCarta 3.0. Lysosomal compartment annotation was performed using combined lysosomal proteome datasets^57–59^. Proteins capable of S-acylation were annotated with our previously published proteome of S-acylated proteins isolated from WT and PPT1 KO 2-month brain and synaptosomes^7^.

LFQ-MS data are available through the ProteomeXchange Consortium^97^ via the PRIDE partner repository^98^ with accession PXD054766. Annotated proteomic data are available in **Data S2**.

### Time-of-Flight Secondary Ion Mass Spectrometry (ToF-SIMS)

WT-12 and PPT1–7 pure lipofuscin fractions (*n =* 3 biological replicates) were diluted with ultrapure distilled H_2_O (RPI) and pelleted, then washed in ultrapure distilled H_2_O to remove sucrose and protease inhibitors (6000 x g, 10 min, 4°C). The washed lipofuscin pellet was frozen at −80°C prior to analysis by ToF-SIMS.

Silicon wafers (5×5 mm, Agar Scientific Limited) were cleaned in a sonication bath with two successive washes in methanol and water (20 min. each), air drying, UV-Ozone cleaning (30 min using Ossila Ltd, UK, model L2002A2-UK), and a final wash in water (20 min) then methanol (20 min). Clean wafers were stored in a glass vial containing 100% methanol. Directly prior to use, silicon wafers were removed from methanol and allowed to air dry.

Washed lipofuscin pellets stored at −80°C were thawed and spread onto silicon wafers with a sterile spatula. The wafers were attached to a custom-made sample holder stub (Ionoptika Ltd, UK) with conductive carbon tape and transferred into a J105 3D Chemical Imager (Ionoptika Ltd, UK)^99^ through a vacuum lock system. The instrument was equipped with both a 40 keV C_60_ ion gun and a 70 keV gas cluster ion beam (GCIB) gun. GCIB gun was used to produce large water clusters with an average size of approximately 19500 water molecules. This large cluster size was used to reduce the amount of fragmentation and improve the molecular ion yield, as previously described^100,101^. The primary ion dose for water clusters was 5.20×10^12^ ions/cm^2^ per layer of analysis. A total of 20 consecutive layers were collected with the same ion dose. A sample area of 200×200 μm was analyzed and collected using 32×32 pixels. The water cluster spot size/lateral resolution was between 5.6 and 9.2 μm. The three biological replicates per genotype were analyzed in technical quadruplicate (water) in both positive and negative ion mode.

ToF-SIMS spectral data files for each of the 20 layers per analyzed area were converted to a MATLAB file format using ChiToolBox (Alex Henderson, University of Manchester; https://github.com/AlexHenderson/ChiToolbox). The data for each layer was summed to represent the total ion intensity across the whole depth profile and to reduce the experimental uncertainty caused by choosing a specific layer to analyze. The whole mass spectrum was averaged over 6 (negative mode) or 12 (positive mode) replicate analyses. The averaged spectra were further processed by taking the square root of the intensities and normalizing the data using Euclidean norm (p-norm where *p* = 2). Pure lipofuscin ToF-SIMS spectra were plotted using GraphPad Prism version 10.0.2 for Windows 64-bit (GraphPad Software, Boston, Massachusetts USA).

### ToF-SIMS of *in situ* lipofuscin

PPT1–7 and WT-12 mice were sacrificed and brains excised as described above for lipofuscin purification. Brains were cut into sagittal hemispheres and embedded in 10% sodium carboxymethyl cellulose resin (419338, Sigma) then snap frozen on dry ice and maintained at −80°C until sectioning. Thin sagittal sections (10 *μ*m) containing the hippocampus were alternately cut with a cryostat (Leica CM1850). Alternate sections were thaw-mounted onto microscope slides coated with 2% gelatin from porcine skin (G1890, Sigma-Aldrich) with 0.1% chromium (III) potassium sulfate dodecahydrate (243361, Sigma-Aldrich) for fluorescence microscopy or onto indium tin oxide (ITO; 576352, Sigma-Aldrich) coated slides for ToF-SIMS. Immediately after sectioning, slides were placed in a glass chamber and a low vacuum was applied. Slides were stored at −80°C prior to ToF-SIMS analysis.

Imaging experiments were conducted using a J105 3D Chemical Imager (Ionoptika Ltd, UK) with a large water cluster ion beam (approximately H_2_O_(29600)_) in positive ion mode. The ion beam had a spatial resolution of 5.6 μm, and a primary ion dose of 5.31×10¹³ ions/cm² was applied to the brain sample. Image processing was performed using Analyse Software (v2.0.2.15; Ionoptika Ltd, UK), with the data subsequently converted to MATLAB (MathWorks) file format. ChiToolbox (Alex Henderson, University of Manchester; https://github.com/AlexHenderson/ChiToolbox) was utilized to open the images in MATLAB and to perform multivariate statistical analysis. Principal component analysis (PCA) and K-means clustering were applied to distinguish different areas of the hippocampus and surrounding brain regions. Pre-processing steps included square rooting, vector normalization, and peak picking prior to multivariate analysis.

### Lipidomics

Whole brain homogenates and purified lipofuscin from PPT1–7 and WT-24 (*n =* 3 biological replicates) were spiked with standards from Avanti Polar Lipids (UltimateSPLASH One (330820), Cardiolipin Mix 1 (LM6003), and Cholesterol-d7 (LM4100)) and extracted using a methyl-tert-butyl-ether separation, as described previously ^102^. Samples were resuspended in 1-BuOH:IPA:H_2_O 8:23:69 and loaded onto an Agilent Poroshell 120 (EC-C18 2.7 *μ*m, 1,000 bar, 2.1 × 100 mM) column using a ThermoFisher Scientific Vanquish Neo UHPLC. The gradient consisted of mobile phase A (60% acetonitrile, 40% H_2_O, 7.5 mM ammonium acetate, 0.1% Formic Acid) and mobile phase B (90% IPA, 10% acetonitrile, 7.5 mM ammonium acetate, 0.1% Formic Acid). A flow rate of 50 uL/min was used to separate peaks for detection on a Thermo Scientific Orbitrap Exploris 240 mass spectrometer. The gradient started at 7% B, increased to 10% B over 0.5 minutes, to 30% B over 1.5 minutes, 40% B over 8 minutes, 55% B over 10 minutes, 60% B over 20 minutes and then to 99.5 % B over 30 minutes. The gradient was then held for 20 min before being restored to 7% and equilibrated. Lipids and standards were identified using MSDIAL 5.2 ^89^. The molar concentrations of each lipid in the sample were normalized to the molar amount of the closest retention time standard for that lipid class.

### PPT1 enzyme activity

PPT1 enzyme activity was determined by the standard assay outlined by van Diggelen et al., 1999 ^103^. Briefly, whole brain homogenates or purified lipofuscin (5 *μ*L) were diluted to 10 *μ*L in McIlvain’s phosphate/citrate buffer, pH 4.0. 20 *μ*L of PPT1 substrate reaction solution (0.64 mM 4-methylumbelliferyl-6-thio-Palmitate-β-D-glucopyranoside (MU-6S-Palm-βGlc; Biosynth, EM06650), 15 mM dithiothreitol (DTT), 0.375% (w/v) Triton X-100, and 0.1 U β-glucosidase from almonds (Sigma-Aldrich, 49290) in McIlvain’s phosphate/citrate buffer, pH 4.0) were added to each sample. PPT1 substrate reaction solution and reaction solution without MU-6S-Palm-βGlc were each added to 10 *μ*L McIlvaine’s phosphate/citrate buffer alone as background controls. Samples were incubated for 1 hour at 37°C. Reactions were terminated by the addition of 200 *μ*L 0.5 M NaHCO_3_/0.5 M Na_2_CO_3_, pH 10.5 with 0.025% (w/v) Triton X-100. Released 4-methylumbelliferone fluorescence was measured in a black, clear-bottom 96-well plate on a BioTek Gen5 Microplate Reader (Agilent) at an excitation of 380 nm and an emission of 454 nm. Activity values were normalized to the highest background control and to total sample concentration (*μ*g) as determined by bicinchoninic acid assay (BCA). To determine specific PPT1 activity, values were further normalized to PPT1 protein levels in the assayed samples, as determined by quantitative immunoblotting after normalizing to beta-actin as a loading control.

### Cathepsin-D enzyme activity

Cathepsin-D fluorometric reactions were prepared using a commercial kit (Abcam ab65302) and measured on a BioTek Gen5 Microplate Reader (Agilent) at an excitation of 328 nm and emission of 460 nm. To obtain Cathepsin-D enzyme activity per *μ*g of brain tissue, fluorescence enzyme activity values were normalized to total brain homogenate concentration as determined by BCA. To obtain specific Cathepsin-D enzyme activity, total cathepsin-D protein (Abcam ab207549) levels were determined by quantitative immunoblotting and normalized to beta-actin as a loading control.

### TPP1 enzyme activity

TPP1 enzyme activity was determined by a method modified from Lukacs et al., 2003^104^. Briefly, 20 *μ*L of TPP1 substrate (300 *μ*M Ala-Ala-Phe-7-amido-4-methylcoumarin (Sigma-Aldrich, A3401) in reaction buffer (acetate buffer with 0.9% NaCL, 10 mM pepstatin A, 56 mM E-64) was added to whole brain homogenates or purified lipofuscin (5 *μ*L). 20 *μ*L of TPP1 substrate reaction solution or reaction solution without substrate were included as background controls. Samples were incubated for 1 hour at 37°C. Reactions were terminated by the addition of 200 *μ*L 0.5 M NaHCO_3_/0.5 M Na_2_CO_3_, 0.1 M glycine, pH 9.7. Released 4-methylcoumarin fluorescence was measured in a black, clear-bottom 96-well plate on a BioTek Gen5 Microplate Reader (Agilent) at an excitation of 355 nm and an emission of 460 nm. Activity values were normalized to the highest background control and to total sample concentration (*μ*g) as determined by BCA.

### GBA1 enzyme activity

GBA1 enzyme activity was determined using an established assay ^105^ that was previously modified to improve accuracy ^106^ and adapted for this study. Briefly, 2 *μ*L of whole brain homogenates were prepared on ice with 23 *μ*L of GBA1 substrate (0.4M 4-methylumbelliferyl-b-D-glucoside (4-MU; CarboSynth EM05983)) in McIlvaine’s phosphate/citric acid buffer, pH 5.1 (0.2 M Na_2_HPO4, 0.1 M citric acid) with 0.01% (w/v) bovine serum albumin (BSA) and 10 *μ*M of the GBA2 inhibitor N-butyldeoxynojirimycin (NB-DNJ; Sigma-Aldrich, B-8299). 23 *μ*L GBA1 substrate reaction solution alone was included as a background control. Samples were incubated for 1 hour at 37°C. The reaction was terminated by the addition of 200 *μ*L cold 0.5 M glycine-NaOH, pH 10.6. Released 4-methylumbelliferyl fluorescence was measured in a black, clear-bottom 96-well plate on a BioTek Gen5 Microplate Reader (Agilent) at an excitation of 366 nm and an emission of 445 nm. Activity values were normalized to the background control and to total sample concentration (*μ*g) as determined by BCA.

### Quantification and statistical analysis

Statistical tests performed for each dataset are described in figure legends. Biological replicates (n) for each experiment are listed in figure legends. Throughout, values are expressed as mean ± standard deviation (SD). *p*-values of 0.05 or less were considered statistically significant and are expressed as horizontal bars with or without asterisks where applicable (* *p* < 0.05; ** *p* < 0.01, *** *p* < 0.001). Further details concerning statistical tests, including specific *p*-values, are detailed in **Data S1**. Fluorescent image data and electron microscopy data were masked to the investigator during analysis. Excel (Microsoft) and GraphPad Prism (v10.2.3) were used to conduct statistical analyses.

## Figures and Tables

**Table 1.** Key resources

REAGENT OR RESOURCE	SOURCE	IDENTIFIER
**Antibodies**		
Mouse anti-ACTB (WB 1:1000)	GeneTex	GTX629630; RRID: AB_2728648
Mouse anti-ATPB (WB 1:1000)	Abcam	ab14730; RRID: AB_301438
Rabbit anti-CATD (IHC 1:500; ICC 1:100)	Abcam	ab75852; RRID: AB_1523267
Rabbit Anti-CATD (WB 1:1000)	Abcam	ab207549; RRID: AB_1523267
Rat anti-LAMP1 (IHC 1:200; ICC 1:200; WB 1:1000)	Developmental Studies Hybridoma Bank (UIowa)	1D4B; RRID: AB_528127
Rabbit anti-LC3 (WB 1:1000)	Sigma	L7543; RRID: AB_796155
Chicken anti-MAP2 (ICC 1:500)	Millipore Sigma	AB5543; RRID: AB_571049
Rabbit anti-MDHM (WB 1:20,000)	Abcam	ab181873; RRID; AB_2893443
Rabbit anti-PPT1 (WB 1:100)	Laboratory of Sreeganga Chandra; Gorenberg et al.^7^	AB2860 EXT
Rabbit anti-PSAP (WB 1:1000)	Abcam	ab300469; RIID: AB_3101805
Mouse anti-SQSTM (WB 1:1000)	Novus Biologicals	H00008878-M01; RRID: AB_548364
Mouse anti-SYPH (WB 1:10,000)	Synaptic Systems	SYS-101-011; RRID: AB_887824
Rabbit anti-SYUA (WB 1:1000)	Cell Signaling	D37A6; RRID: AB_1904156
2° Goat anti-Rabbit IgG Alexa Fluor 488 (ICC 1:500)	Invitrogen	A-11008; RRID: AB_143165
2° Goat anti-Rat IgG Alexa Fluor 568 (ICC 1:500)	Invitrogen	A-11077; RRID: AB_2534121
2° Goat anti-Rabbit IgG Alexa Fluor 633 (IHC 1:500)	Invitrogen	A-21070; RRID: AB_2535731
2° Goat anti-Rat IgG Alexa Fluor 633 (IHC 1:500)	Invitrogen	A-21094; RRID: AB_2535749
2° Goat anti-Chicken IgY Alexa Fluor 647 (ICC 1:500)	Invitrogen	A-21449; RRID: AB_2535866
2° Goat anti-Mouse IgG 680 RD (WB 1:6000)	Licor	926-68070
2° Goat anti-Rabbit IgG 800 CW (WB 1:6000)	Licor	926-32211
**Biological samples**		
Human dorsolateral prefrontal cortex sections	Center for Human Brain Discovery Brain Bank, Yale Pathology Department	BDBB-1
**Chemicals, peptides, and recombinant proteins**		
Isoflurane	Henry Schein Animal Health	NDC 11695-6776-2
Heparin sodium salt	Sigma-Aldrich	H3393
Paraformaldehyde (PFA)	Sigma-Aldrich	158127
Tissue-Tek OCT medium	Sakura	4583
Gelatin from porcine skin	Sigma-Aldrich	G1890
Chromium (III) potassium sulfate dodecahydrate	Sigma-Aldrich	243361
Antifade mounting medium with DAPI	Vectashield	H-1200
Phosphate Buffered Saline (PBS)	Gibco	10010023
Triton X-100	Sigma-Aldrich	X100
Normal Goat Serum (NGS)	Gibco	16210064
Sucrose	Sigma-Aldrich	S0389
N-[2-Hydroxyethyl] piperazine-N′-[2-ethanesulfonic acid] (HEPES)	American Bio	AB00892
Phenylmethylsulfonyl fluoride (PMSF)	American Bio	AB01620
Aprotinin	Sigma-Aldrich	A6279
Leupeptin	American Bio	AB01108
Pepstatin A	Thermo Scientific	78436
complete, Mini, EDTA-free Protease Inhibitor Cocktail Tablets	Roche	11873580001
Solvent Black 3 (SB3)	Sigma-Aldrich	199664
Antifade mounting medium	Vectashield	H-1000
2% Uranyl acetate	Electron Microscopy Sciences	22400
2.5% Glutaraldehyde	Electron Microscopy Sciences	16537
0.1M Sodium cacodylate, pH 7.4	Electron Microscopy Sciences	11650
1% Osmium tetroxide	Electron Microscopy Sciences	19140
Propylene oxide	Electron Microscopy Sciences	20401
EPON 812 epoxy resin	Electron Microscopy Sciences	14900
Reynold’s lead citrate	Electron Microscopy Sciences	22410-01
0.8% Potassium ferrocyanide	Electron Microscopy Sciences	25154
Dulbecco’s Modified Eagle Medium (DMEM)	Gibco	11965-092
Fetal bovine serum (FBS)	Gibco	A56708-01
Penicillin/streptomycin	Gibco	15140-122
Seahorse XF DMEM	Agilent	103575-100
Glucose	Agilent	103577
Glutamine	Agilent	103579
L-carnitine	Millipore Sigma	C0283; CAS 664-46-1
Bovine serum albumin (BSA)	Agilent	102720-100
Hoescht 33342	Thermo Fisher	62249
RapiGest SF	Waters Corporation	186001861
Ammonium bicarbonate (NH_4_HCO_3_)	Millipore Sigma	A6141; CAS 1066-33-7
Dithiothreitol (DTT)	Millipore Sigma	3860-OP; CAS 3483-12-3
PNGase	Promega	V4831
Lys-C	Thermo Fisher Scientific	90307
Trypsin	Thermo Fisher Scientific	90305
Trifluoroacetic acid (TFA)	Thermo Fisher Scientific	28901
Formic acid (FA)	Thermo Fisher Scientific	28905
Acetonitrile (ACN)	Thermo Fisher Scientific	047138.M1
10X Pierce Retention Time Calibration Mixture	Thermo Fisher Scientific	88321
UltimateSPLASH One	Avanti Polar Lipids	330820
Cardiolipin Mix 1	Avanti Polar Lipids	LM6003
Cholesterol-d7	Avanti Polar Lipids	LM4100
4-methylumbelliferyl-6-thio-Palmitate-β-D-glucopyranoside (MU-6S-Palm-βGlc)	Biosynth	EM06650
β-glucosidase from almonds	Sigma-Aldrich	49290
Ala-Ala-Phe-7-amido-4-methylcoumarin	Sigma-Aldrich	A3401
E-64	Thermo Scientific	78434
Sodium bicarbonate (NaHCO_3_)	Sigma-Aldrich	S5761; CAS 144-55-8
Sodium carbonate (Na_2_CO_3_)	Sigma-Aldrich	223530; CAS 497-19-8
Glycine	Fisher Scientific	BP381
4-methylumbelliferyl-b-D-glucoside (4-MU)	CarboSynth	EM05983
Sodium phosphate dibasic (Na_2_HPO_4_)	Sigma-Aldrich	S9763; CAS 7558-79-4
Citric acid	Sigma-Aldrich	251275; CAS 77-92-9
N-butyldeoxynojirimycin (NB-DNJ)	Sigma-Aldrich	B-8299
**Critical commercial assays**		
QIAquick PCR Purification Kit	Qiagen	28104
GenePORTER 3000 Transfection Reagent	amsbio	AMS.T203007
Seahorse Cell Mito Stress Test	Agilent	103015-100
Cathepsin-D Enzyme Activity Kit	Abcam	ab65302
Bicinchoninic acid (BCA) Protein Assay Kit	Thermo Scientific	23235
Seahorse XF Cell Mito Stress Test Kit	Agilent	103015-100
**Deposited data**		
QUINT lipofuscin atlas data	This paper	https://lipofuscinatlas.yale.edu/
Raw western blot images	This paper	Mendeley: DOI: 10.17632/y55sncg5s4.1
Raw proteomics data	This paper	PRIDE: PXD054766
**Experimental models: Cell lines**		
HEK293T	ATCC	CRL-3216
HEK293T-PPT1-KO	This paper	N/A
**Experimental models: Organisms/strains**		
C57BL/6J	The Jackson Laboratory	000664; RRID: IMSR_JAX:000664
B6;129-*Ppt1^tm1Hof^*/J	The Jackson Laboratory; Gupta et al.^9^	004313; RRID: IMSR_JAX:004313
**Oligonucleotides**		
CRISPR target sequence for *PPT1:* CACCGCGCCGCTGCCGTTGGTGATC	This paper	N/A
U6 promoter primer: GACTATCATATGCTTACCGT	Weinberg Lab; Addgene	LKO.1 5′
*PPT1* forward primer: TTTTGATTCACCGCAGAGGG	This paper	N/A
*PPT1* reverse primer 1: ATCCCATGCCAGATCACCAA	This paper	N/A
*PPT1* reverse primer 2: GTAAAACTTCAACGCCGTGC	This paper	N/A
**Recombinant DNA**		
pSpCas9(BB)-2A-GFP (PX458)	Addgene; Ran et al.^83^	48138
**Software and code**		
QuPath (version 0.02.3)	Bankhead et al.^84^	RRID :SCR_018257; https://qupath.github.io
QuickNII-ABAMouse-v3 2017	Puchades et al.^85^	https://www.nitrc.org/frs/?group_id=1341
VisuAlign (version 0_9)	Maja A. Puchades & Jan G. Bjaalie	RRID:SCR_017978; https://github.com/HumanBrainProject/VisuAlign
Nutil (version 0.8.0)	Groeneboom et al.^86^	RRID:SCR_017183; https://www.nitrc.org/projects/nutil/
Mouse-Brain-Heatmap-Website	Laboratory of Michael Henderson	https://github.com/MXHend/Mouse-Brain-Heatmap-Website?tab=readme-ov-file
NutilToUsable	Laboratory of Michael Henderson	https://github.com/DaniellaDeWeerd/NutilToUsable
Mouse_Brain_Heatmap	Allen Institute; Wang et al.^87^	https://github.com/vari-bbc/Mouse_Brain_Heatmap
Allen Mouse Brain Atlas CCFv3	Allen Institute; Wang et al.^87^	RRID:SCR_020999; http://atlas.brain-map.org
FCS Express (version 7)	De Novo Software	RRID :SCR_016431; https://denovosoftware.com
Image Studio (version 5.2.5)	LICOR	RRID:SCR_015795; https://www.licor.com/bio/image-studio/
Excel (version 16.92)	Microsoft	RRID:SCR_016137; https://www.microsoft.com/en-us/microsoft-365/excel
FIJI ImageJ (version 214.0/1.54f)	Schindelin et al.^88^	RRID:SCR_002285; https://fiji.sc
GraphPad Prism (version 10.1.1 for mac; version 10.0.2 for Windows 64-bit)	GraphPad Software Inc.	RRID:SCR_002798; https://www.graphpad.com
Progenesis QI (version 4.2)	Waters Nonlinear Dynamics	RRID:SCR_018923; https://www.nonlinear.com/progenesis/qi-for-proteomics
Mascot search engine (version 2.7)	Matrix Science	RRID:SCR_000307; https://www.matrixscience.com
Ingenuity Pathway Analysis (IPA) (version 1.23.01)	QIAGEN Inc.	RRID:SCR_008653; https://digitalinsights.qiagen.com/IPA
MS-DIAL (version 5.2)	Tsugawa et al.^89^	RRID:SCR_023076; https://github.com/systemsomicslab/MsdialWorkbench/releases
Analyse (version 2.0.2.15)	Ionoptika Ltd	https://ionoptika.com/news/analyse-v2-0-2-15-release/
MATLAB (version R2024a)	The MathWorks Inc.	RRID:SCR_001622; https://www.mathworks.com/products/matlab
ChiToolBox	Alex Henderson, University of Manchester	https://github.com/AlexHenderson/ChiToolbox
**Other**		
Flow cytometry sub-micron particle size reference kit	Thermo Fisher Scientific	F13839
Seahorse XFe/96/XF Pro 96-well microplates	Agilent	103794-100
Seahorse XFe96/XF Pro FluxPak	Agilent	103793-100
Silicon wafers (5×5 mm)	Agar Scientific Limited	AGG3390
Indium tin oxide (ITO) coated slides	Sigma-Aldrich	576352

## Data Availability

Lipofuscin brain atlas data are available as a web tool at: https://lipofuscinatlas.yale.edu/. Raw mass spectrometry data are available through the ProteomeXchange Consortium via the PRIDE partner repository with accession PXD054766. Note: Reviewers can access these data with the username: reviewer_pxd054766@ebi.ac.uk and password: 4nwzFfYubcv9. Numerical source data underlying graphical figures are found in **Data S1**. Annotated proteomic data are found in **Data S2**, and Ingenuity Pathway Analysis data are found in **Data S3**. Original western blot images have been deposited at Mendeley at DOI: 10.17632/y55sncg5s4.1 and are publicly available as of the date of publication. Microscopy data reported in this paper will be shared by the lead contact upon request. Any additional information required to reanalyze the data reported in this paper are available from the lead contact upon request.
